# In‐Cell Characterization of the Stable Tyrosyl Radical in *E. coli* Ribonucleotide Reductase Using Advanced EPR Spectroscopy

**DOI:** 10.1002/anie.202102914

**Published:** 2021-06-04

**Authors:** Shari L. Meichsner, Yury Kutin, Müge Kasanmascheff

**Affiliations:** ^1^ Department of Chemistry and Chemical Biology TU Dortmund University Otto-Hahn-Strasse 6 44227 Dortmund Germany

**Keywords:** EPR spectroscopy, metalloenzymes, ribonucleotide reductase, tyrosyl radicals, unnatural amino acids

## Abstract

The E. coli ribonucleotide reductase (RNR), a paradigm for class Ia enzymes including human RNR, catalyzes the biosynthesis of DNA building blocks and requires a di‐iron tyrosyl radical (Y_122_^.^) cofactor for activity. The knowledge on the in vitro Y_122_^.^ structure and its radical distribution within the β2 subunit has accumulated over the years; yet little information exists on the in vivo Y_122_^.^. Here, we characterize this essential radical in whole cells. Multi‐frequency EPR and electron‐nuclear double resonance (ENDOR) demonstrate that the structure and electrostatic environment of Y_122_^.^ are identical under in vivo and in vitro conditions. Pulsed dipolar EPR experiments shed light on a distinct in vivo Y_122_^.^ per β2 distribution, supporting the key role of Y^.^ concentrations in regulating RNR activity. Additionally, we spectroscopically verify the generation of an unnatural amino acid radical, F_3_Y_122_^.^, in whole cells, providing a crucial step towards unique insights into the RNR catalysis under physiological conditions.

## Graphical abstract

The in vivo structure of the redox‐active tyrosyl radical in ribonucleotide reductase that catalyses the biosynthesis of DNA building blocks was explored using advanced EPR spectroscopic techniques. Furthermore, the radical form of a tyrosine derivative within the cells was generated. Our results mark the initial steps towards gaining unique insights into the redox reactions involving tyrosyl radicals under physiological conditions.

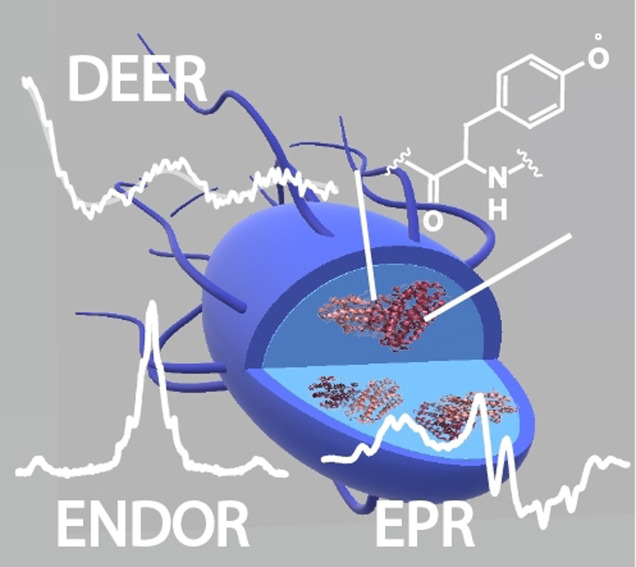

## Introduction

Ribonucleotide reductases (RNRs) catalyse the conversion of nucleotides to their corresponding deoxynucleotides in every living cell.^[1]^ Due to their central role in DNA replication and repair, they have been a target for cancer therapeutics.^[2]^ *E. coli* class Ia RNR, a paradigm for class Ia enzymes including human RNR, consists of two homodimeric subunits, α2 and β2. The active α2β2 complex is formed transiently upon substrate and effector binding.^[3]^ The catalytic reaction of *E. coli* class Ia RNR relies on at least five redox‐active amino acids, which are involved in an unprecedented reversible radical transfer that occurs over ≈32 Å.^[4]^ β harbours the stable di‐iron tyrosyl radical cofactor Y_122_^.^ and the redox‐active tyrosine residue Y_356_. α contains the active site for nucleotide reduction, two allosteric effector binding sites and redox active residues Y_731_, Y_730_, and C_439_ that are a part of the radical transfer pathway. Y_122_^.^ reversibly oxidizes C_439_ to a thiyl radical, which in turn initiates the irreversible catalytic reaction.

As di‐iron tyrosyl radical cofactor initiates the catalytic reaction, radical generation at this tyrosine residue is essential for RNR activity. Y^.^ is generated by self‐assembly requiring Fe^2+^ and O_2_, both in structurally homogenous *E. coli* and *Saccharomyces cerevisiae* (*S. cerevisiae*) class Ia RNRs.^[5]^ However, radical generation efficiency varies among different organisms and studies (in vivo vs. in vitro).[^2b^, ^5^] In vitro studies with *E. coli* RNR showed that usually 1.2 Y^.^/wt‐β2 were generated, suggesting ≈60 % of the β2 population is active and ≈40 % possesses inactive diferric clusters without any Y^.^s (“two or none” radical distribution model).^[6]^ In vivo Y^.^ levels, however, were substoichiometric compared to that of iron loading.^[7]^ Furthermore, in vivo radical distribution in *E. coli* (one or two radicals per β2) remains unknown. In contrast, studies with *S. cerevisiae* revealed stoichiometric amounts of Y^.^ with 1 Y^.^ per ββ′ heterodimer both in vivo and in vitro.[^5^, ^8^] These studies together demonstrated that *S. cerevisiae* RNR activity is not regulated by the modulation of Y^.^ concentrations, but Y^.^ concentrations play a key role in regulation of *E. coli* RNR activity in cells.

The rate‐limiting step in the *E. coli* RNR catalytic reaction, which is the conformational change(s) upon substrate and allosteric effector binding, has prevented spectroscopic detection of other redox‐active amino acids in wild‐type enzyme.^[9]^ The involvement of Y^.^ intermediates in RNR catalytic reaction was evidenced by site‐specific incorporation of unnatural amino acids (UAAs) with altered p*K*_a_s and reduction potentials such as DOPA, NH_2_Y, NO_2_Y, and F_*n*_Ys.^[10]^ Radical formation on these UAAs not only allowed characterization of tight α2β2 complex but also provided valuable insights into proton‐coupled electron transfer steps within RNR via transient absorption^[11]^ and advanced electron paramagnetic resonance (EPR) spectroscopies.^[12]^ For example, distance measurements conducted using double electron‐electron resonance (DEER, also known as PELDOR)^[13]^ combined with incorporation of UAAs provided structural constraints for redox‐active pathway tyrosine residues and displayed an asymmetry within the α2β2 complex.[^10c^, ^14^] Same strategy was further used to gain insights into the relative redox potentials of three subsequent pathway tyrosyl radicals,^[10g]^ and to detect the flexibility of pathway residue Y_731_ in the α2β2 complex for the first time.^[12b]^ Very recently, near‐atomic‐resolution cryo‐EM structure of the active α2β2 complex, which is asymmetric, was achieved by employing F_3_Y_122_^.^.^[4a]^

**Figure 1:**
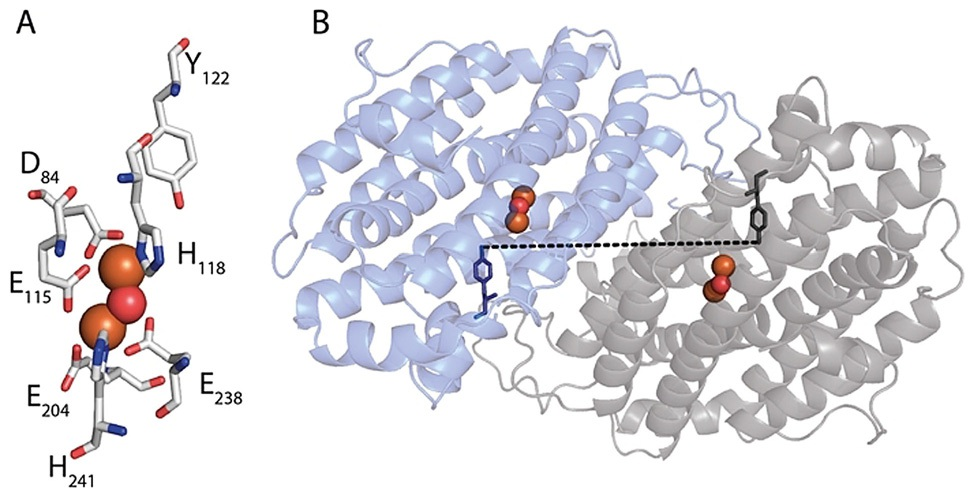
A) Environment of Y_122_ in the β2 subunit of *E. coli* RNR. Y_122_ (grey sticks) is located next to a di‐iron centre (Fe: orange‐ and O: red‐sphere), which is ligated by six amino acids (white sticks). B) The distance measured in this work is shown with a dashed line in the homodimeric β2 subunit (PDB 5CI4).

One of the most challenging tasks for techniques used in structural biology is to achieve high resolution within the native environment of biomolecules, that is, the cells. Among the methods employed, EPR spectroscopy is an emerging tool for in vivo studies.^[15]^ It is the natural choice for investigating biomolecules with intrinsic paramagnetic centres, and it has been central to our understanding of not only RNRs but also other fundamental enzymes such as hydrogenases, nitrogenases and photosystem II.^[16]^ Here, we have generated Y_122_^.^, the stable di‐iron tyrosyl radical cofactor of *E. coli* class Ia RNR in whole cells. We characterized its in‐cell conformation and H‐bonding environment at nanometre‐scale resolution using multi‐frequency EPR and electron‐nuclear double resonance (ENDOR) spectroscopies. Subsequently, we detected in vivo distances between two Y_122_^.^s by DEER (Figure 1). These orientation‐selective experiments revealed insights into not only the in vivo structure and dynamics of Y_122_^.^ and β2 subunit but also the in vivo radical distribution within β2. This first example of distance measurements between two native paramagnetic centres of an enzyme within intact cells where the protein under investigation is expressed adds another dimension to similar attempts wherein a spin label is directly introduced in the cells.^[17]^ Additionally, we have site‐specifically incorporated 2,3,5‐trifluorotyrosine (F_3_Y) at residue 122, generated and identified its radical form F_3_Y_122_^.^ in the cells, and obtained in vivo distances between F_3_Y_122_^.^s. These results verify the generation of an unnatural amino acid radical in whole cells and show that F_3_Y incorporation does not alter protein structure within cells.

## Results and Discussion

### Generation, Detection, and Characterization of Y_122_^.^ in Cells

Presence of the di‐iron centre is prerequisite for the radical state of Y_122_ and for the catalytic activity of *E. coli* RNR. Previously, it was shown that in vitro incubation of iron‐free, apo‐β2 with Fe^II^ and O_2_ resulted in the reconstitution of the radical state of Y_122_, and thus led to recovery of the RNR activity.^[18]^ Here, we extend this technique to in vivo conditions. Apo‐β2 in *E. coli* was over‐expressed because Y_122_ concentrations are below detection limit under normal growth conditions.^[7]^ Cell cultures were harvested, washed several times and resuspended in a buffer containing Fe^II^ for 10 min. Next, the cell suspension was saturated with O_2_ and transferred into EPR tubes (Supporting Information (SI) 1.1). Prior to EPR experiments, the viability of the cells was checked using cell counting experiments (SI 1.3). These proved that the cells used for EPR measurements were intact and alive. In addition, the possibility of cell damage, and thus high amounts of protein leakage from the cells into the suspension buffer was ruled out using polyacrylamide gel electrophoresis and EPR experiments (SI 2.1).

**Figure 2:**
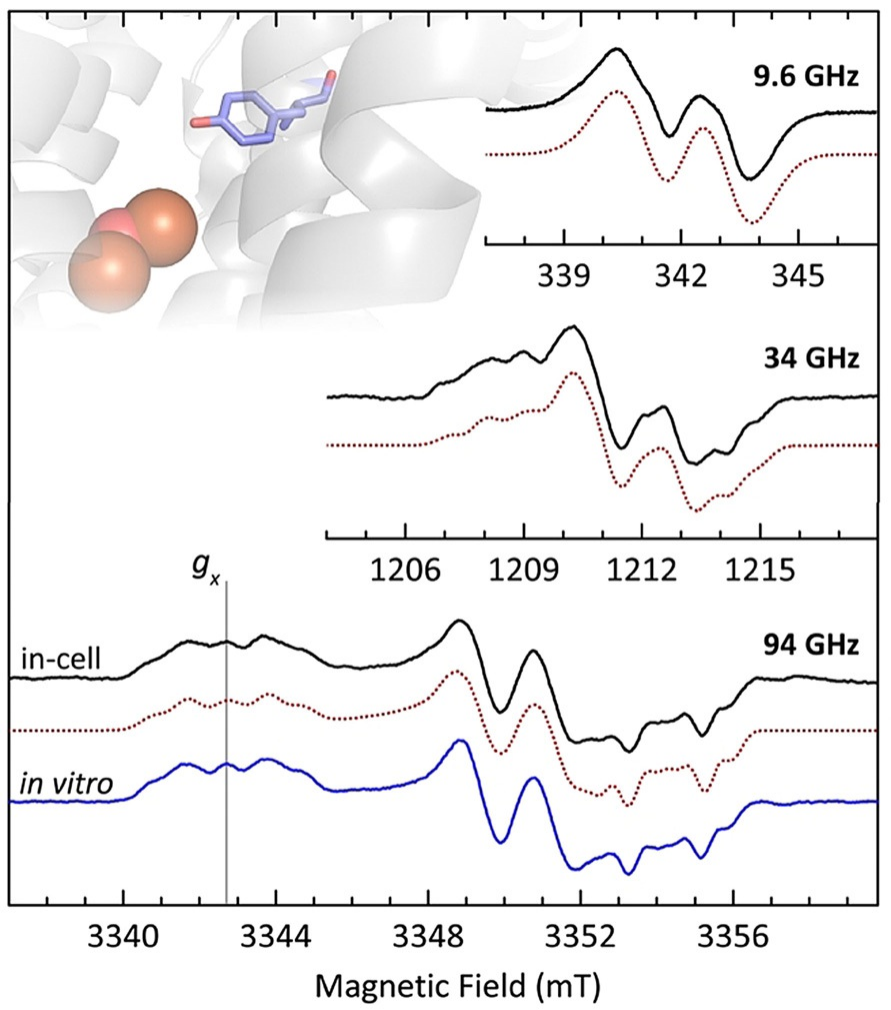
Continuous wave EPR (9.6 GHz, top) and first‐derivative pulse EPR (34 GHz, middle and 94 GHz, bottom) spectra of Y_122_^.^ in whole *E. coli* cells are shown in solid black lines along with the corresponding EasySpin^[23]^ simulations in dotted red lines. See SI 2.6 for the background correction details. The 94 GHz EPR spectrum of in vitro Y_122_^.^ (solid blue line) is shown for comparison. Position of the *g*_x_ value is displayed with a grey vertical line. Inset: The isolated di‐iron centre (orange and red spheres) and residue Y_122_ (blue sticks) are shown within the protein environment.

Comparison of 9.6 GHz EPR spectra of treated and untreated whole *E. coli* cells showed that the Fe^II^ and O_2_ treatment led to the generation of a radical species whose EPR signature is similar to that of Y_122_^.^ (SI 2.2). To examine the generated radical, we recorded multi‐frequency (9.6, 34 and 94 GHz) EPR spectra of treated whole *E. coli* cells (termed in‐cell) and analysed the data using spectral simulations (Figure 2). Principal *g* values and hyperfine coupling parameters related to ring‐ and β‐methylene protons reported for *E. coli* Y_122_^.^ under in vitro conditions (Table S3 and Scheme S1) were in excellent agreement with the multi‐frequency EPR/ENDOR dataset.^[19]^ These results showed that the radical generated within cells is Y_122_^.^ having an average bulk concentration of 18±5 μm (see SI 2.3 for concentration determination).

94 GHz EPR spectrum of in vitro Y_122_^.^ is also shown in Figure 2 for comparison. At high frequencies the EPR line shape is dominated by *g*‐ and hyperfine anisotropy and is highly sensitive to molecular environment of tyrosyl radicals.[^12d^, ^20^] The *g*_x_ value reports on the electrostatic environment of a tyrosyl radical and is affected by the changes in the radical's local environment, such as addition or loss of an H‐bond.[^12d^, ^20a^, ^21^] Furthermore, distributions in *g*_x_ value indicate multiple molecular orientations and/or radical environments. Another important structural parameter is the strength of the hyperfine couplings of Cβ‐methylene protons. They are related to the dihedral angle between C*β*−H bonds and the ring plane, and therefore provide information on the structure of tyrosyl radicals.^[22]^

Therefore, a substantial change in the structure of Y_122_^.^ would result in a different β‐methylene proton hyperfine coupling, and thus distinct EPR line shape (SI 2.4), as it has been observed previously for residue Y_731_.^[12b]^ Here, we detected a *g*_x_ value for in‐cell Y_122_^.^ that is identical to that of in vitro one, resulting in nearly identical EPR line shapes for in‐cell and in vitro Y_122_^.^. The only marginal difference observed in the line shapes is due to the Mn^2+^ species that is inherent to the cells (see SI 2.5 and 2.6, as well as a comparison of two in‐cell W‐band spectra without background correction in Figure S4 B, SI 2.4). Overall, all of the EPR data and simulations clearly show that Y_122_^.^ generated within the cells is a single radical species with one set of magnetic parameters and a well‐defined orientation. Additionally, our results demonstrated that structure and environment of Y_122_^.^ in the cells are highly similar to those of Y_122_^.^ in vitro. These results were not unexpected considering the isolated nature of Y_122_^.^ inside the protein, around 10 Å away from its surface.

### H‐Bonding Environment of Y_122_^.^ in Cells

**Figure 3:**
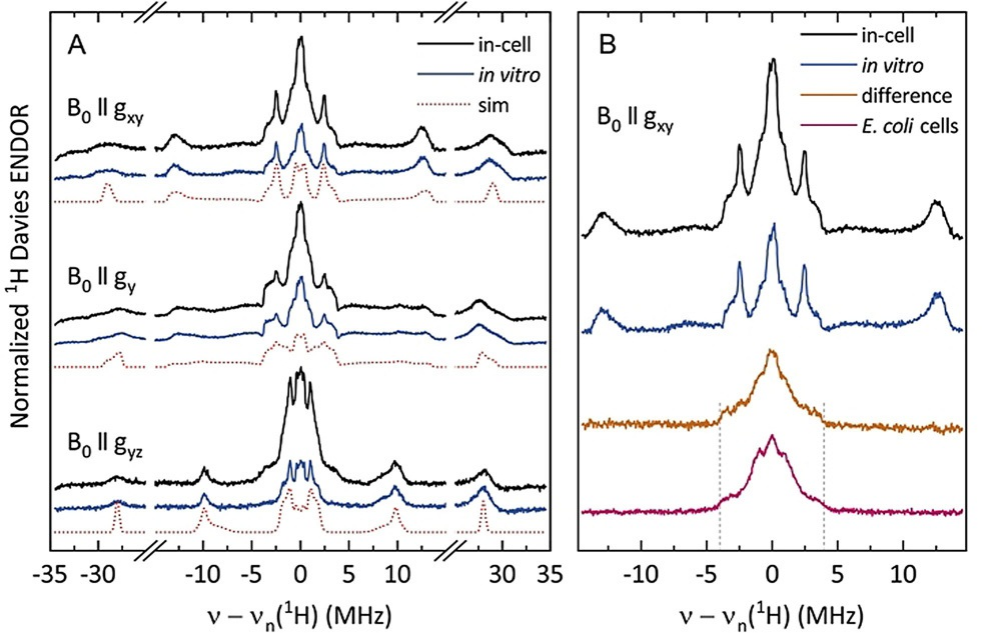
A) Orientation‐selective ^1^H Davies ENDOR spectra of in‐cell (black) and in vitro (blue) Y_122_^.^ recorded at 34 GHz at three field positions corresponding to *g*=2.0094 (*B*_0_ II *g*_xy_), 2.0059 (*B*_0_ II *g*_y_), and 2.0005 (*B*_0_ II *g*_yz_). Simulations (red dotted lines) were performed using the spin Hamiltonian parameters determined in Ref. 18c (Table S3). B) ENDOR spectra (*B*_0_ II *g*_xy_) of the in‐cell (black) and in vitro (blue) Y_122_^.^, the scaled subtraction result (orange), and Mn^2+^‐related ^1^H ENDOR features of *E. coli* cells not treated with iron (purple). Dashed vertical lines indicate spectral features associated with [Mn(H_2_O)_6_]^2+^.

Next, we employed ENDOR spectroscopy at 34 GHz to probe the H‐bonding environment of Y_122_^.^ in the cells. Orientation‐selective ENDOR reports on the number, separation distance (usually ≤15 Å) and orientation of the magnetic nuclei that are coupled to the observed radical, and thus provides structural information.^[24]^ We recorded ENDOR spectra at three field positions within the Y_122_^.^ EPR line corresponding to *g*_xy_, *g*_y_ and *g*_yz_ molecular orientations shown in SI 2.6. Figure 3 A illustrates the orientation‐selective ^1^H Davies ENDOR spectra of Y_122_^.^ in the cells (black lines) and in vitro (blue lines) with the corresponding simulations (red dotted lines). Based on the previous ^1^H ENDOR studies on Y_122_^.^ conducted at 9 up to 263 GHz,[^19c^, ^25^] seven internal ^1^H hyperfine couplings were included in the simulations of the in vitro and in‐cell spectra (see Table S3 and Scheme S1). As expected, the simulations were in excellent agreement with in vitro ENDOR and multi‐frequency EPR data.

The ^1^H Davies ENDOR line shape of the in‐cell Y_122_^.^ contained additional spectral features compared to the in vitro Y_122_^.^. Therefore, the in vitro spectrum was subtracted from the in‐cell one after normalizing the spectra to the C_1_β‐methylene proton ENDOR intensities (couplings around ±28 MHz in Figure 3 A). The resulting spectrum (orange trace in Figure 3 B) is very similar to the ^1^H Davies ENDOR spectrum recorded for *E.coli* cells that contain overexpressed apo‐β2 without tyrosyl radicals (labeled *E. coli* cells, purple trace). This comparison clearly demonstrated that the additional proton hyperfine couplings observed for the in‐cell sample are not related to Y_122_^.^, but rather to Mn^2+^ in *E. coli* cells as the only clearly detectable paramagnetic species in these cells was Mn^2+^ (SI 2.6). Indeed, the additional ^1^H ENDOR features detected in *E. coli* cells closely resemble the in‐cell Mn^2+^ ENDOR line shape found in literature,^[26]^ with the broader shoulders reaching ±3.8 MHz around the ^1^H Larmor frequency (dashed vertical lines) indicative of the water‐ligated Mn^2+^ species.^[27]^ We note that the assignment of additional proton hyperfine couplings to the Mn^2+^ species is in line with identical *g*_x_ values detected for in‐cell and in vitro Y_122_^.^. An extra H‐bond to Y_122_^.^ would make its local environment more polar and shift its *g*_x_ to lower values, as reported previously for tyrosyl radicals of distinct proteins coupled to hydrogens of similar strengths.[^12d^, ^21^] Furthermore, the ^1^H hyperfine coupling observed in the difference spectrum, which is similar to the *A*_y_ component of C_3_/C_5_ protons, would result in additional splittings in the 94 GHz EPR spectrum as shown in Figure S4, which were not observed. Overall, the analysis of our orientation‐selective ^1^H Davies ENDOR data combined with the *g*_x_ value and EPR line shape information demonstrated that the number of hydrogens coupled to Y_122_^.^ as well as the coupling strengths are the same in the cells and in vitro.

### In‐Cell Distance and Radical Distribution Information Obtained Using DEER Spectroscopy

**Figure 4:**
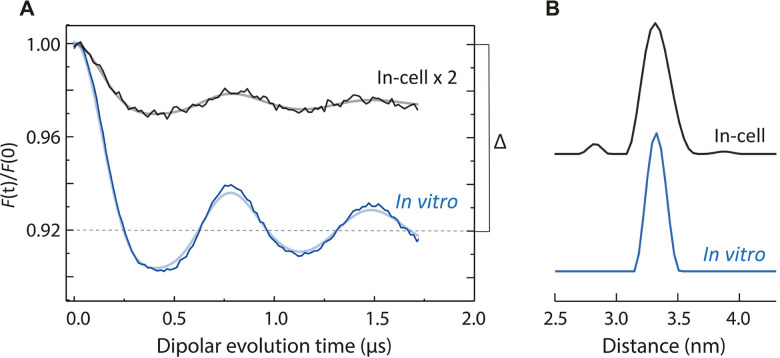
Background‐ and phase‐corrected, normalized and orientation‐averaged 34 GHz in‐cell (black) and in vitro (blue) DEER traces of β2 subunit of *E. coli* RNR (A) shown along with obtained Y_122_^.^‐Y_122_^.^ distance distributions (B). DEER time traces were analysed with DeerAnalysis2019^[32]^ and the fits are overlaid in grey (in‐cell) and light blue (in vitro). Details of the experiments and analyses are given in SI 2.7. The original in‐cell trace was magnified by a factor of two for better visualization.

In order to get insights into the wt‐β2 structure and in‐cell radical distribution, we measured the distance between two Y_122_^.^′s, each residing in a β monomer of *E. coli* RNR in whole cells (Figure 4). As orientation selectivity, which occurs when dipolar spectrum is dependent on the selected *g*‐tensor orientations,^[28]^ was reported previously for the in vitro Y_122_^.^‐Y_122_^.^ pair,^[29]^ we employed orientation‐selective DEER spectroscopy. Three DEER traces were recorded at distinct *g*‐tensor orientations covering the whole EPR line, and the obtained dipolar traces were summed (see SI 2.7). Fourier‐transform of in‐cell and in vitro summed traces led to almost ideal Pake patterns (SI 2.7). The resulting orientation‐averaged form factors and the extracted distance distributions are displayed in Figure 4. The analysis of the in‐cell DEER data revealed a main mean distance at 3.32 nm with a distance distribution of *σ*=0.09 nm (σ is the standard deviation of the distribution, which is assumed Gaussian). This distance belongs to the in‐cell Y_122_^.^‐Y_122_^.^ pairs, as it is identical to in vitro Y_122_^.^‐Y_122_^.^ distances detected in this work (3.32 nm with *σ*=0.07 nm) and previously (3.31 nm^[30]^). An additional peak with an extremely small intensity emerged from the analysis of the in‐cell data. It is most likely an artifact caused by the orientation‐averaging procedure due to low signal‐to‐noise ratios (SNRs) of individual in‐cell DEER traces, as well as the overlap between Y_122_^.^ and Mn^2+^ EPR features (see SI 2.8 for details). The detected narrow in‐cell distance distribution (σ<0.1 nm) and the presence of orientation selectivity demonstrated the rigid nature of Y_122_^.^s, and more importantly, of the β2 subunit within the cells. This conclusion is also supported by our EPR data that established Y_122_^.^ generated within the cells as a single and structurally defined radical species.

**Figure 5:**
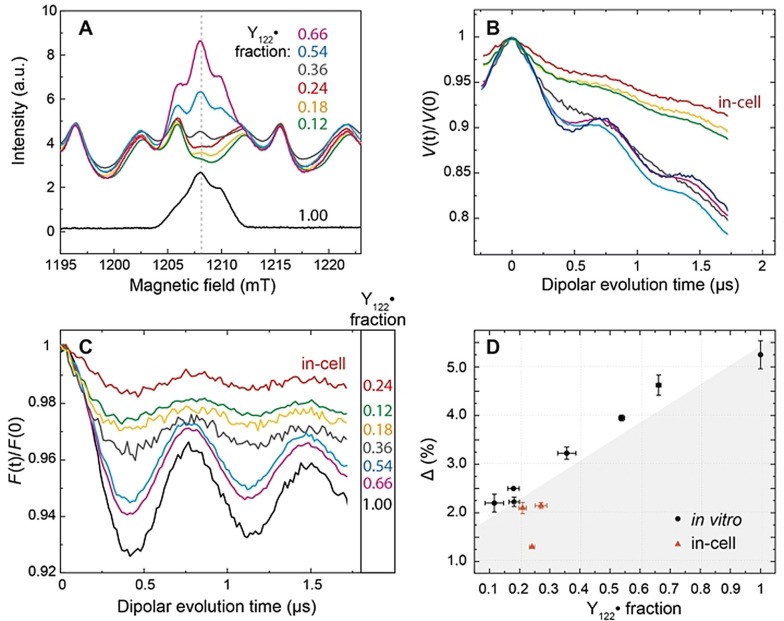
Modulation depth analysis of in vitro β2 samples containing different equivalents of Mn^2+^ (see Table S2 for sample specifications). A) Q‐band field‐swept EPR spectra of RNR/Mn^2+^ mixtures and an in‐cell sample (red trace) recorded using refocused spin echo with the pump pulse applied at the primary echo position. The importance of the pump pulse in refocused echo experiments involving a high‐spin species has been reported previously.^[33]^ The spectra were normalized to the Mn^2+^ intensity. B) Primary DEER traces and C) form factors of the corresponding samples (see SI 2.11 for details of the background correction). Y_122_^.^ fractions were calculated as I(Y_122_^.^)/ [I(Mn^2+^) + I(Y_122_^.^)] at the observe position (dashed line in A). D) Calibration curve for the modulation depth as a function of relative Y_122_^.^ contribution to the refocused echo. Area below the curve is shaded as a guide to the eye. For details, see SI 2.9 and 2.10.

Being able to detect a Y_122_^.^‐Y_122_^.^ distance in the cells is not self‐evident because in vivo radical distribution of β2 is not known. In order to investigate the radical distribution within the cells, which was suggested to play a key role in RNR regulation and activity,[^2b^, ^7^] we analysed the modulation depth parameter Δ (shown in Figure 4 A for the in vitro trace). Δ delivers information on the fraction of spin pairs in a sample, and is affected by the presence of monomers and other paramagnetic species.^[31]^ Comparison of DEER traces recorded at the same orientation and under identical experimental conditions showed that Δ values of in‐cell samples were significantly lower than that of the in vitro one (1.3–2.1 % vs. 5.3 %, see Figure S10, as well as the red and black traces in Figure 5 C). A strong reduction in Δ for the in‐cell samples was expected, given the large contribution from Mn^2+^ to the EPR signal at the observe position (SI 2.5 and Figure 5 A). Note that the variations observed in the in‐cell modulation depths were batch‐dependent despite the identical sample preparation procedure (Figure S10). This outcome was not surprising given the number of various factors that could affect cell growth and were not controlled under our experimental conditions.

To decipher whether Mn^2+^ solely accounts for the reduction of Δ in the cells, we quantified its effect on Δ by adding various amounts of Mn^2+^ to in vitro samples (Figure 5, see Table S2 for details). As expected, the resulting calibration curve, which shows Δ as a function of relative Y_122_^.^ contribution to the refocused echo, revealed a clear reduction in the Δ values with the increasing Mn^2+^ fraction (Figure 5 C/D and SI 2.10 for details of the analysis). Δ of the in vitro wt‐β2 samples without Mn^2+^ depends neither on the protein nor on the spin concentration of the sample,^[29]^ as wt‐β2 dimer in vitro either contains a Y_122_^.^‐Y_122_^.^ pair or no radical at all (“two or none” model^[6a]^). Therefore, Mn^2+^ is the only reason for the reduced Δ of the in vitro samples. We note that Mn^2+^ ions are homogeneously distributed, and thus do not contribute to the detected distances and distributions throughout this work (Figure S9).

As seen in Figure 5 D, the in‐cell Δ values (red data points) fall consistently below the in vitro calibration curve (grey shaded area). This result suggests that the reduction of Δ in the cells cannot be explained by the presence of Mn^2+^ alone. As Y_122_^.^ and Mn^2+^ were the only paramagnetic centres detected within the cells (Figures 2, S5, and S6), we conclude that some of the β2 dimers present in the cells contain only one Y_122_^.^, further reducing the in‐cell Δ values. In vivo presence of 1 Y^.^ per β2 is consistent with the asymmetric α2β2 complex structure.^[8]^ Although the in‐cell fraction of β2 subunits having only one tyrosyl radical could be calculated based on the fit resulted from the calibration curve, we refrain from such an analysis at this point. A detailed and controlled investigation of cell growth conditions such as growth time, added solutes, metal inhibition should be done to determine the exact in vivo monomeric 1 Y^.^/β2 fraction, which is beyond the scope of this work and will be addressed in future studies. Overall, our data suggest a distinct radical distribution in *E. coli* cells compared to the in vitro “two or none” model, and to that of in vivo *S. cerevisiae*.

Additionally, we compared background slopes of the DEER dipolar traces, as the slope reports on the local spin concentrations in a sample.^[34]^ The background steepness of in‐cell samples was highly similar to that of in vitro samples with the radical concentration of 22 μm and 5–6 equivalents of added Mn^2+^ (red vs. yellow and green traces in Figure 5 B, see Table S2). In vitro DEER traces for samples with higher radical concentrations (70 and 150 μm) showed significantly steeper backgrounds (grey, blue, pink, and black traces in Figure 5 B). This demonstrated that the local Y_122_^.^ concentration within the cells was substantially lower than 70 μm, and was similar to that of the in vitro sample, which was prepared to mimic the in‐cell sample and contained 22 μm Y_122_^.^ (yellow trace in Figure 5 B and SI 2.12). This result is in very good agreement with the intracellular Y_122_^.^ concentration of around 20–32 μm as determined by spin quantification at 9.6 GHz (SI 2.3). The detection of low intracellular Y_122_^.^ concentration is further supported by echo decay measurements. *T*_m_ of Y_122_^.^ within cells is either longer or the same as that of in vitro samples (see Figure S8).

### Detection of F_3_Y_122_^.^ and F_3_Y_122_^.^‐ F_3_Y_122_^.^ Pairs in Cells

Incorporation of F_3_Y_122_^.^ was fundamental to being able to investigate in vitro radical transfer mechanism in RNR and to obtain the cryo‐EM structure of the α2β2 complex. Therefore, we explored the possibility of observing F_3_Y^.^ in whole *E. coli* cells. Successful generation of F_3_Y_122_^.^ within the cells would be the first step towards gaining unique insights into the RNR catalysis under physiological conditions. In addition, it may serve as a new exciting probe to study in vivo radical transfer steps in other fundamental proteins containing amino acid radicals.

In order to generate F_3_Y_122_^.^, we followed the same procedure as that described for the in‐cell generation of Y_122_^.^. After over‐expressing apo‐F_3_Y_122_‐β2 in the cells, we treated the harvested and washed cell cultures with Fe^II^ and O_2_ to generate F_3_Y_122_^.^ (SI 1.2). Prior to EPR experiments, the viability of the cells and protein leakage from the cells were checked using cell counting and EPR experiments, respectively (SI 1.3 and 2.13). The resulting data showed that the cells used for EPR measurements were intact and no detectable amounts of F_3_Y_122_^.^‐β2 protein pass through the *E. coli* cell wall. 9.6 and 34 GHz EPR measurements along with spectral simulations revealed the presence of the F_3_Y_122_^.^ species in the cells (SI 2.13). Spin quantification experiments yielded a 17±5 μm in‐cell sample concentration of F_3_Y_122_^.^ (SI 2.13), in good agreement with that of the wt‐β2 in‐cell samples.

**Figure 6:**
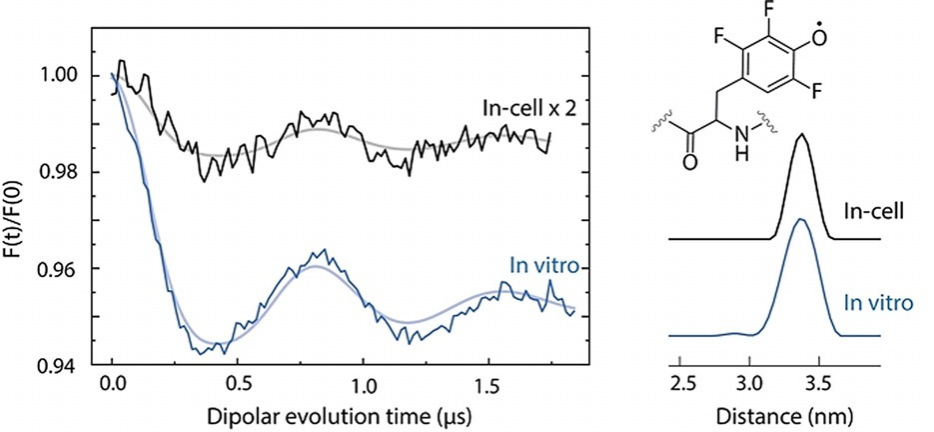
Background‐ and phase‐corrected, normalized 34 GHz in‐cell (black) and orientation‐averaged in vitro (blue) DEER traces of F_3_Y_122_^.^‐β2 subunit of *E. coli* RNR (A) shown along with obtained F_3_Y_122_^.^‐F_3_Y_122_^.^ distance distributions (B). The structure of F_3_Y^.^ is shown in the inset (B). DEER time traces were analysed by DeerAnalysis2019^[32]^ and the fits are overlaid in grey (in‐cell) and light blue (in vitro). Details of the experiments and analyses are given in SI 2.14. The original in‐cell trace was magnified by a factor of two for better visualization.

Subsequently, in‐cell and in vitro DEER measurements with samples containing F_3_Y_122_^.^ were performed (Figure 6). Four in vitro DEER traces recorded at different *g*‐tensor orientations were summed to eliminate orientation‐selection effects (see SI 2.14 for details). Such orientation‐selective DEER measurements were not feasible with the in‐cell sample because of poor SNR. We recorded an in‐cell DEER trace at a molecular orientation at which the distance vector ***r***_F3Y122‐F3Y122_ is perpendicular to the magnetic field because the correct mean distances can be directly obtained from dipolar frequencies at this orientation.^[35]^ The distance analysis of this trace resulted in a mean distance of 3.37 nm (standard deviation of the in‐cell distance distribution, σ, which is not totally reliable in the presence of orientation selectivity,^[32]^ is not given). The detected in‐cell distance is assigned to a F_3_Y_122_^.^‐F_3_Y_122_^.^ pair as it agrees extremely well with the in vitro distance detected here (3.36 nm with *σ*=0.11 nm) and with the distance between oxygen atoms of F_3_Y_122_′s in the recent crystal structure of F_3_Y_122_‐β2 (SI 2.15).^[36]^

Furthermore, we compared background steepness of in‐cell and in vitro F_3_Y_122_^.^ DEER traces. Similar to the results obtained with Y_122_^.^, this analysis showed that local F_3_Y_122_^.^ concentration within the cells is substantially lower than the detected in vitro concentration of 130±30 μm (SI 2.13 for spin quantification, and SI 2.14 for background comparison).

## Conclusion

This study has revealed that the structure and electrostatic environment of the essential di‐iron tyrosyl radical cofactor Y_122_^.^ in *E. coli* RNR are identical under in vivo and in vitro conditions. Here, we presented distance measurements between two native paramagnetic centres residing in an enzyme in intact and living cells where the protein under investigation is expressed. Distance measurements within the Y_122_^.^‐Y_122_^.^ pair provided insights into the in‐cell structure and conformational rigidity of the β2 subunit, and suggested a distinct in‐cell radical distribution within this subunit. Detection of β2 subunits having only one Y_122_^.^ strongly supports a model in which *E. coli* RNR activity is regulated by modulation of Y^.^ concentrations in the cells.^[7]^ Our results serve as a basis for future experiments aimed at detecting and manipulating the factors influencing in‐cell RNR activity. Additionally, we present spectroscopic detection of an unnatural amino acid radical in whole cells, and show that F_3_Y incorporation does not affect the in vivo protein structure. Successful generation of F_3_Y_122_^.^ within the cells is the first step towards gaining unique insights into the RNR catalysis under physiological conditions. Furthermore, it showcases the possibility of unravelling the in vivo structure and role of tyrosyl radicals involved in other fundamental processes, such as photosynthesis, reduction of O_2_ to water, and DNA repair by unnatural amino acid incorporation.

## Conflict of interest

The authors declare no conflict of interest.

## Supporting information

As a service to our authors and readers, this journal provides supporting information supplied by the authors. Such materials are peer reviewed and may be re‐organized for online delivery, but are not copy‐edited or typeset. Technical support issues arising from supporting information (other than missing files) should be addressed to the authors.

Supporting Information
