# Supplementary material for: In‐Cell Characterization of the Stable Tyrosyl Radical in E. coli Ribonucleotide Reductase Using Advanced EPR Spectroscopy
Source: Angew Chem Int Ed Engl. 2021 Jun 4;60(35):19155–61. doi: 10.1002/anie.202102914 (PMC8453577; doi:10.1002/anie.202102914)
Supplement: Supplementary file 1 — Supporting Information [file ANIE-60-19155-s001.pdf]

Angewandte Chemie
Eine Zeitschrift der Gesellschaft Deutscher Chemiker

Supporting Information

In-Cell Characterization of the Stable Tyrosyl Radical in *E. coli* Ribonucleotide Reductase Using Advanced EPR Spectroscopy

*Shari L. Meichsner, Yury Kutin, and Müge Kasanmascheff**

anie_202102914_sm_miscellaneous_information.pdf

Table of Contents

1. Experimental Procedures ... 3

1.1. Wild-type protein expression, purification and preparation of EPR samples ... 3

1.2. (2,3,5)F_3Y_{122}-β2 protein expression, purification and preparation of EPR samples ... 3

1.3. Cell counting experiments ... 3

1.4. Spectrometer specifications, sample concentrations and details of the EPR experiments ... 4

2. Results and Discussion ... 6

2.1. SDS-PAGE gel electrophoresis and EPR analysis to determine possible protein leakage ... 6

2.2. 9.6 GHz EPR spectra of treated and untreated whole *E. coli* cells ... 7

2.3. Estimation of in-cell spin concentration ... 9

2.4. Hyperfine coupling pattern of Y_{122}• shown along with its 94 GHz EPR spectra ... 10

2.5. Detection and evidence of Mn^{2+} species in the cellular environment ... 11

2.6. Subtraction of the Mn^{2+} spectral features present in the EPR spectrum of an in-cell sample. Details of orientation-selective ^{1}H ENDOR spectra recorded at 34 GHz ... 12

2.7. Details of the Y_{122}• DEER measurements and analysis ... 13

2.8. Origin of the second distance observed with in-cell sample ... 16

2.9. DEER data of three distinct in-cell samples ... 17

2.10. Calibration curve for determining the expected modulation depths of in-cell samples ... 18

2.11. Background correction details ... 19

2.12. Concentration determination of the *in vitro* mimic sample ... 21

2.13. Detection of F_3Y_{122}• in whole *E. coli* cells ... 22

2.14. Details of the F_3Y_{122}• DEER measurements and analysis ... 24

2.15. F_3Y_{122}-F_3Y_{122} distance obtained from the crystal structure of F_3Y_{122}-β2 ... 26

References ... 27

1. Experimental Procedures

1.1. Wild-type protein expression, purification and preparation of EPR samples

E. coli BL21(DE3)-Gold (Invitrogen) were transformed with pTB-*nrdB*, which encodes for wt-β2, and plated on LB-agar plates with 100 µg/mL carbenicillin (Carb) at 37 °C. Positive clones were selected and a starter culture (5 mL) was grown overnight in LB-medium enriched with Carb at 37 °C until saturation. An intermediate culture (100 mL) was enriched with 1 mL of the starter culture, also grown overnight. The expression culture was grown with a 200-fold dilution of the intermediate culture in LB-medium containing Carb. At OD600 ~ 0.9, 1,10-Phenanthroline was added to a final concentration of 100 µM. After 20 min, protein expression was induced with 0.5 mM IPTG. Apo-β2 in *E. coli* was over-expressed because β2 endogenous expression level is far below the EPR detection limit[1] (see also Figure S2B). After 4 h of protein overproduction, the cells were harvested by gentle centrifugation. Although 7000 g for 20 min centrifugation was performed for *E.coli* whole-cell RNR experiments previously[1], here the centrifugation is performed at 6000 g for 15 min at 4 °C in order to keep as many cells as possible intact and alive. A typical 2.5-3 g cell paste/L media was obtained, in great agreement with previously reported yield considering 1,10-Phenanthroline addition.[1] For *in-vitro* sample preparations, the apo-β2 construct was purified by anion-exchange chromatography and radical was generated as previously described.[2] Protein concentration was determined via UV-vis spectroscopy. 200 µL protein solution was transferred to EPR tubes and shock frozen in liquid N_2. For in-cell sample preparations, the harvested cell pellet was washed several times and resuspended in 50 mM Tris pH 7.6 with 5% glycerol using 3 mL of buffer per g cell paste. 0.13 mM $Fe^{II}(NH_4)_2(SO_4)_2$ (~5 equivalents of Fe^{II} with respect to the estimated protein concentration) was added to this buffer. The added Fe concentration was only 13 % of that, which is shown to inhibit *E. coli* cell growth.[3] The suspension was allowed to sit on ice for 10 min and subsequently saturated with O_2 gas on ice for 1 – 2 min. 200 µL cell-suspension was directly transferred into EPR-tubes and frozen gently in an isopropanol rack at -80 °C to secure slow cooling and prevent cell damage. Spin concentrations of the samples are determined via 9.6 GHz cw-EPR measurements (see SI 2.3).

1.2. (2,3,5)F_3Y_{122}-β2 protein expression, purification and preparation of EPR samples

E. coli BL21(D3)-Gold cells (Invitrogen) were co-transformed with pBAD-*nrdB*$_{122TAG}$ and pEVOL-F_nYRS-E3, and plated on LB-agar plates with 100 µg/mL carbenicillin (Carb) and 35 µg/mL chloramphenicol (Cm) at 37 °C. Positive clones were selected and a starter culture (5 mL) was grown overnight in LB-medium enriched with Carb and Cm at 37 °C until saturation. An intermediate culture (100 mL) was enriched with 1 mL of the starter culture, also grown overnight. The expression culture was grown with a 200-fold dilution of the intermediate culture in 2XYT medium containing Carb and Cm. At OD600 ~ 0.3, F_3Y was added to a final concentration of 0.7 mM. After 30 min, 100 µM 1,10-phenanthroline was added to chelate iron. After further 30 min, protein expression was induced with 0.5% (w/v) L-arabinose. After 4 h protein overproduction, the cells were harvested by gentle centrifugation at 6000 g for 15 min at 4°C. For *in-vitro* sample preparations, the apo-(2,3,5)F_3Y_{122} construct was purified by anion-exchange chromatography and radical was generated as previously described.[4] Protein concentration was determined via UV-vis spectroscopy. 200 µL protein solution was transferred to EPR tubes and shock frozen in liquid N_2. For in-cell sample preparations, the harvested cell pellet was washed several times and resuspended in 50 mM Tris pH 7.6 with 5% glycerol using 3 mL of buffer per g cell paste. 0.13 mM $Fe^{II}(NH_4)_2(SO_4)_2$ (~5 equivalents of Fe^{II} with respect to the estimated protein concentration) was added to this buffer. The BL21 cell-suspension was allowed to sit on ice for 5 min and afterwards saturated with O_2 gas on ice for 1 – 2 min. 200 µL of cell-suspension was directly transferred into EPR-tubes and frozen gently in an isopropanol rack at -80 °C to secure slow cooling and prevent cell damage.

1.3. Cell counting experiments

Prior to EPR experiments, four distinct cell suspensions were plated on LB-agar plates after 1:10^{12} (1:10^{11} for (2,3,5)F_3Y_{122} construct) dilution to check the viability of the cells. The cell growth on plates with the *E. coli* cell suspensions that did not contain any overexpression plasmid were 3 – 13 · 10^{11} and 4 · 10^{11} cells per mL cell suspension before and after iron addition, respectively. The cell growth on the plates with the *E. coli* cell suspension containing wt-β2 overexpression plasmid, which is used for EPR experiments, was 3 – 60 · 10^{11} cells per mL cell suspension, in great agreement with previously reported numbers.[1] The cell growth on the plates with the *E. coli* cell suspension containing (2,3,5)F_3Y_{122} overexpression plasmid, which is used for EPR experiments, was at least 6 · 10^{10} cells per mL cell suspension. These experiments proved that the cells used for EPR measurements were intact and alive.

1.4. Spectrometer specifications, sample concentrations and details of the EPR experiments

X-band: X-band cw-EPR measurements were carried out at T = 100 K using a Bruker EMX-Nano Benchtop spectrometer equipped with a continuous-flow nitrogen cryostat.

Q-band: Q-Band pulse EPR measurements were carried out at T = 10 K using a Bruker Elexsys E580 spectrometer equipped with a 150 W TWT amplifier, Bruker ER 5106QT-2 resonator, Bruker SpinJet AWG, Oxford Instruments CF935 continuous-flow helium cryostat and Oxford Instruments MercuryiTC temperature controller.

Orientation-selective DEER experiments were performed using the following dead-time free 4-pulse DEER pulse sequence: $\pi/2_{obs}$-τ1-π_{obs}-(τ1+T)-π_{pump}-(τ2-T)-π_{obs}-τ2-echo with artifact-free 16-step phase cycling[5] and Gaussian pulses.[6] The frequency separation, $\Delta f = f_{pump} - f_{obs}$, was 84 MHz, and an overcoupled resonator with f_{pump} set to the center of the resonator dip was used. The optimal π-pulse lengths were determined using transient nutation experiments and were typically ~30 ns for the pump pulse and ~70 ns for the detection. DEER time traces were background-corrected by using an empirical second-order polynomial fitting, if not stated otherwise. For orientation-averaging a recently reported procedure was used.[7] Each primary time trace was first normalized to the same signal intensity at zero time. Afterwards, these traces were normalized to the signal intensity at the pump position. Summation of the DEER traces led to the orientation-averaged time trace. Spin-concentrations: 240 µM (*in vitro*), 22 µM (in-cell).

ENDOR experiments were carried out at 10 K using a Bruker EN 5107D2 resonator and an AR 600 W radiofrequency (RF) amplifier (AR 600A225A). Orientation-selective ^{1}H Davies ENDOR spectra were recorded using the following microwave pulse sequence: π-T-$\pi/2$-τ-π-τ-echo. The RF pulse of variable frequency was applied during the time interval T and had a length of 17 µs. The first π pulse was a rectangular-shaped inversion pulse of 190 ns. Three consecutive measurements at field positions corresponding to g = 2.0094, 2.0059 and 2.0005 were performed for the in-cell and *in vitro* samples. For the sample of *E. coli* cells the ENDOR measurement was performed at g = 2.0094, with the microwave power optimized for a S = 5/2 species. Spin-concentrations: 18 µM (*in vitro*), 14 µM (in-cell).

W-band: W-band pulse EPR measurements were carried out at T = 20 K using the Bruker E680 spectrometer equipped with a Cryogenic Systems closed-cycle 6T magnet and a variable temperature insert (VTI) that allowed varying the temperature within the range 2 – 300 K. Additional LakeShore 335 temperature controller equipped with a Cernox sensor was used to record the sample temperature. Due to a relatively high uncertainty in the external magnetic field values (B_0), the field axis of the W-band EPR spectra was adjusted so that the Y_{122}• spectral width matched the spin Hamiltonian parameters used for the X- and Q-band EPR/ENDOR simulations. This was achieved by compressing the x-axis of all field-swept W-band spectra by ~5%. Spin-concentrations: 18 µM (*in vitro*), 22 µM and 30 µM (in-cell).

Table S1. Acquisition parameters for 34 GHz and 94 GHz Hahn-echo experiments performed in this work are given.

Parameters	34 GHz	94 GHz
**Temperature (K)**	10	20
**π (ns)**	24	32
**τ (ns)**	300	320
**shot repetition time (ms)**	8	15
**shots/point**	100	14 (*in vitro*)
		80 (in-cell 1)
		180 (in-cell 4)
**videogain (dB)**	30	12 (*in vitro*)
		27 (in-cell 1)
		30 (in-cell 4)
**number of scans**	1	1

Table S2. Y• concentrations determined via cw-EPR experiments recorded at X-band and added Mn^{2+} concentrations.

Experiment	Sample	Y• (μM)#	Mn^{2+} (μM)
ENDOR			
	in vitro	18	0
	in-cell 3	14	-
Orientation-selective DEER			
	in vitro	240	0
	in-cell 1	22	-
W-Band EPR			
	in vitro	18	0
	in-cell 1[a]	22	-
	in-cell 4[a]	30	-
Calibration curve			
Y_{122}• fractions as explained in Figure 5:	1	150	0
	0.66	150	100
	0.54	150	200
	0.36	72	280
	0.18 (mimic 1)*	22	100
	0.18 (mimic 2)*	22	100
	0.12	22	132
	in-cell 1[a]	22	-
	in-cell 2[a]	22	-
	in-cell 3[a]	14	-
Experiments with F_3Y_{122}•			
	in vitro	130	0
	in-cell	17	-

#: Radical concentrations are detected via spin quantification experiments at X-band. An inherent error of at most 20 % should be taken into account.
*: Samples were produced from different *in vitro* protein purification batches.
a: Samples were produced from different cell growths, each starting from new LB-agar plates. All samples were prepared in the same way, except for the harvesting centrifugation step. In-cell 1 was centrifuged at 6000 g for 15 min at 4 °C (see SI 1.1), whereas in-cell 2,3 and 4 were centrifuged at 3000 g for 20 min at 4 °C.

2. Results and Discussion

2.1. SDS-PAGE gel electrophoresis and EPR analysis to determine possible protein leakage

In order to detect possible protein leakage that is more than the expected range for *E. coli* cells, experiments were performed with the supernatant of *E. coli* BL21(DE3)-Gold cell pellet collected after wt-β2 expression and Fe^{II} and O_2 treatment. Firstly, SDS-Page analysis was done by comparing the intensity of the collected supernatant with that of *in vitro* wt-β2 protein with known concentrations, namely 2 μM and 5 μM. The intensities of the loaded purified wt-β2 samples were clearly stronger than that of the collected supernatant (see Figure S1A). The comparison of the bands observed in SDS-PAGE gel revealed that the extracellular protein concentration of in-cell sample is < 2 μM. Secondly, the cw-EPR spectrum of the in-cell sample was compared to two supernatants collected from different in-cell samples. Comparison of the cw-EPR spectra demonstrated that the contribution of the extracellular proteins containing radicals is negligible. These results showed that the data collected and analyzed in the present study mostly belong to the radical that resides in wt-β2 in intact *E. coli* cells.

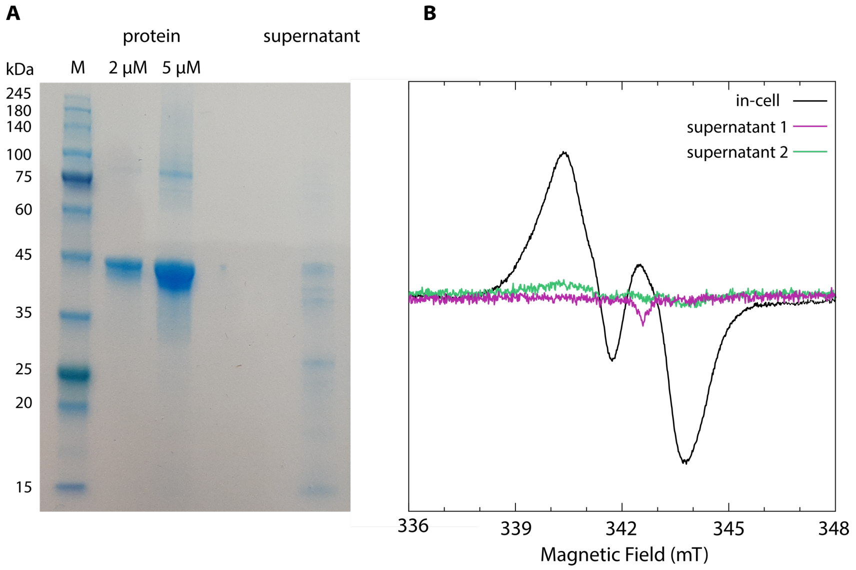

Figure S1. Determination of protein leakage outside the cells via SDS-PAGE (A) and EPR analysis (B). (A) Lane M: molecular mass standards (5 µL); Lane protein: 5 µL of 0.9 and 2 µg purified wt-β2 having a molecular mass of 44 kDa per monomer. The concentrations of the loaded wt-β2 protein correspond to 2 and 5 µM, respectively. Lane supernatant: 5 µL supernatant of *E. coli* BL21(DE3)-Gold cell pellet (containing 14 µM Y_{122}• in total, shown in Figure S3) collected after wt-β2 expression and Fe^{II} and O_2 treatment. (B) Cw-EPR spectra of Y_{122}• recorded at 9.6 GHz and 100 K with Bruker EMXnano. Spectrum of 22 µM wt-β2 expressing whole *E. coli* cells that were prepared as explained in SI 1.1. is shown in black. The supernatant of two samples was collected after centrifugation for 10 min at 2000 g and 4 °C and the resulting spectra are shown in green and purple. Supernatant 1 originates from a 14 µM in-cell sample, also used in (A). The only signal detected for this sample arises from the resonator background. Supernatant 2 was taken from the 22 µM in-cell sample shown in black. Experimental conditions are: 31.6 mW power, 1.5 G modulation amplitude, 100 kHz modulation frequency, 5.12 ms as time constant, and 19.9 ms conversion time, 200 scans (black), 67 scans (purple), 50 scans (green).

2.2. 9.6 GHz EPR spectra of treated and untreated whole *E. coli* cells

Continuous wave (cw) EPR spectra of in-cell samples were recorded before and after treatment with Fe^{II} and O_2 (Figure S2A). The cell pellet was resuspended in 50 mM Tris pH 7.6 with 5% glycerol using 3 mL of buffer per g cell paste. For the treated cells, the buffer contained ~0.13 mM $Fe^{II}(NH_4)_2(SO_4)_2$ additionally and the cell suspension was allowed to incubate on ice for 10 min and afterwards saturated with O_2 gas on ice for 1 – 2 min. In addition, cw-EPR spectra of *E. coli* cells without the wt-β2 expression plasmid pTB-*nrdB* were recorded in order to exclude any signal from the cells (Figure S2B). EPR spectral comparison demonstrates that the treatment led to the generation of a radical species only in the presence of the wt-β2 expression plasmid pTB-*nrdB*, Fe^{II} and O_2.

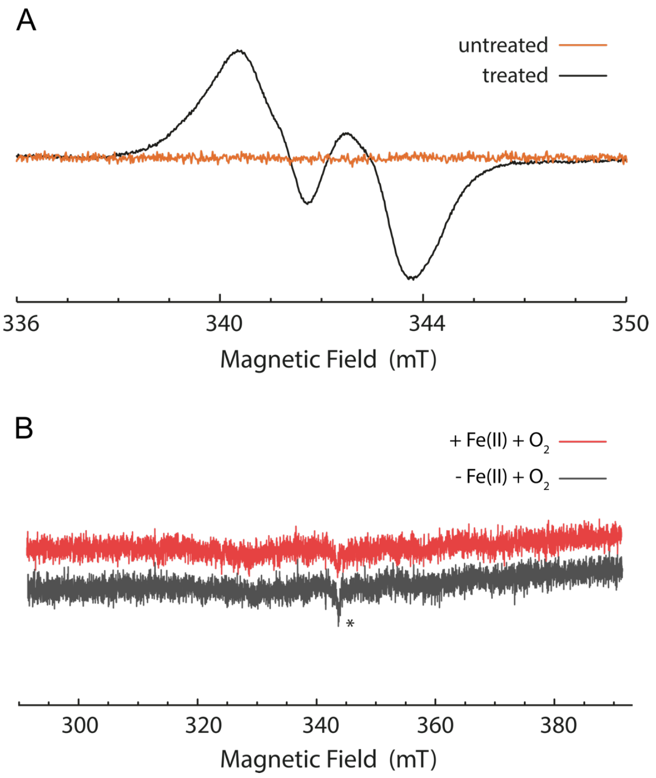

Figure S2. (A) Cw-EPR spectra of wt-β2 expressed in whole *E. coli* cells recorded before (orange) and after (black) Fe^{II} and O_2 treatment. Data were recorded at 9.6 GHz and 100 K with Bruker EMXnano. Experimental conditions: 31.6 mW power, 1.5 G modulation amplitude, 100 kHz modulation frequency, 5.12 ms as time constant, and 19.9 ms conversion time; 40 scans (orange) and 200 scans (black). (B) Cw-EPR spectra of *E. coli* cells grown without wt-β2 expression plasmid recorded at 9.6 GHz and 100 K. Before the EPR experiments, the cells were treated either with Fe^{II} and O_2 or only with O_2. The same experimental conditions as in A); 30 scans (red and black). Signal arising from the resonator background is marked with an asterisk *.

Table S3. ^{1}H Hyperfine coupling parameters used for spectral simulations of the Q-band ENDOR and multi-frequency EPR spectra of Y_{122}• in whole *E. coli* cells, in combination with the g-tensor $g_{x,y,z}$ = 2.00915, 2.00460, 2.00225.[8] The Euler angles α, ß, and γ are defined within the EasySpin z,y',z'' convention. They refer to rotations from the *g*-tensor frame into the hyperfine tensor frames. Positive angles are clockwise rotations viewed along the rotation axis.

	A_x / MHz	A_y / MHz	A_z / MHz	α	β	γ
**$C_1\beta$-H**	59.0	55.0	56.0	0	0	0
**$C_2\beta$-H**	8.0	-1.0	-1.0	70	4	-7
**$C\alpha$-H**	1.2	-1.0	-4.1	60	-20	0
**C_3/C_5 ring protons**	-27.2	-7.9	-19.8	0	0	-25/-155
**C_2/C_6 ring protons**	4.9	7.7	1.7	0	0	25/155

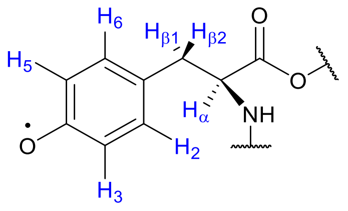

Scheme S1. Schematic representation of Y_{122}• shown with the numbering of protons included into the EPR spectral simulations in blue.

2.3. Estimation of in-cell spin concentration

The continuous wave (cw) EPR signal intensity reports on the spin concentration in a sample.[9] Therefore, we performed cw-EPR experiments with in-cell and *in vitro* wt-β2 samples to estimate in-cell spin concentration. The protein and radical content of the *in vitro* sample were determined as 200 ± 50 μM and 1.2 Y•/protein dimer via UV-vis spectroscopy prior to EPR experiments. Based on these data, we calculated the Y_{122}• concentration as 240 ± 60 μM in the *in vitro* sample. Subsequently, intensities of EPR spectra recorded with five in-cell samples prepared from different growths and/or distinct overexpression levels were compared to that of the *in vitro* sample. This comparison demonstrated that the in-cell radical concentration was ca. 18 μM on average with a maximum variation between batches of ± 6 μM, approximately an order of magnitude lower than that of the 240 μM *in vitro* sample (blue trace in Figure S3). It should be noted that 18 μM is the average bulk concentration in the EPR tube which contains 200 μL of the in-cell sample. In order to estimate the spin concentration within the cells, we calculated the intracellular aqueous volume of BL21(DE3) cells in the EPR tube either as 127 or as 167 μL based on two different methods.[10] As the protein leakage out of the cells was less than 2 μM (see SI 2.1.), the spin concentration within the cells was estimated approximately as 25 ± 4 μM. We note that the highest spin concentration we detected in our in-cell samples with different growth conditions was approximately 22 μM (darkest grey line in Figure S3). This sample has been used for multi-frequency EPR and DEER experiments (named in-cell 1). According to the calculation mentioned above, the intracellular Y_{122}• concentrations of in-cell 1 is 32 ± 6 μM. The sample used for ENDOR experiments (named in-cell 3) showed an intracellular Y_{122}• concentration of 20 ± 4 μM.

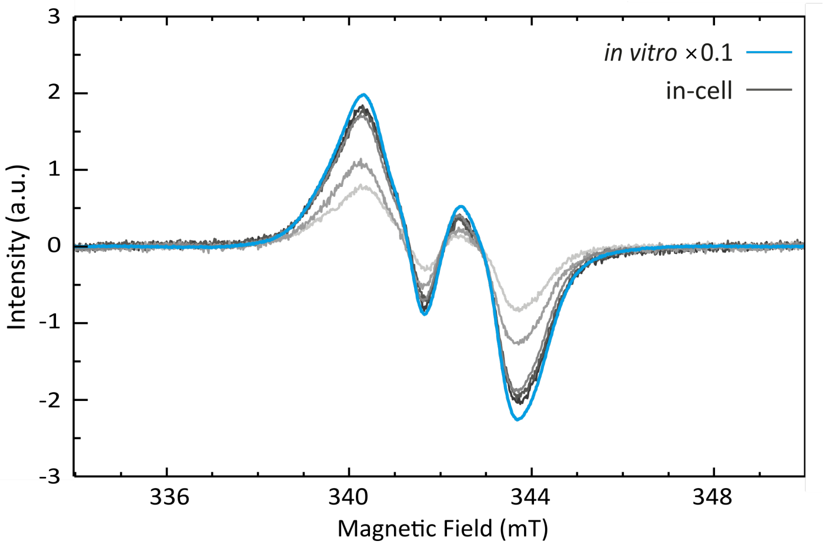

Figure S3. Determination of spin concentration in the whole cells via EPR. Cw-EPR spectra of wt-β2 containing Y_{122}• recorded at 9.6 GHz and 100 K, normalized to the number of averages. Blue: 200 μM purified RNR with 240 μM spin concentration. Grey: Five in-cell wt-β2 samples containing at least 10, at most 22 μM radical (bulk). Data were recorded at the Bruker EMXnano with 31.6 mW power, 1.5 G modulation amplitude, 100 kHz modulation frequency, 5.12 ms as time constant, and 19.9 ms conversion time; grey traces: 50 - 200 scans, blue trace: 20 scans.

2.4. Hyperfine coupling pattern of Y_{122}• shown along with its 94 GHz EPR spectra

The 94 GHz EPR spectrum of Y_{122}• is dominated by *g*- and hyperfine (hf) anisotropy. The rhombic *g*-tensor causes spectral splitting (first level in spectral splitting scheme shown below). The spectrum is further split at the three principal *g*-value positions due to anisotropic hf couplings from magnetically coupled nuclei as listed in Table S3. The *β*-methylene proton (^{1}H-C*β*1) split the lines into two at the principal *g*-values (second level in the spectral splitting scheme shown below). The C_3 and C_5 ring protons are equivalent, and thus they further split the lines into three with signal intensity ratios of 1:2:1 (third level in the spectral splitting scheme). The strongest hf coupling results from the ^{1}H-C*β*1. *β*-methylene proton hf couplings provide information on the structure of tyrosyl radicals via the McConnell equation.[11] Therefore, any change related to ^{1}H-C*β*1 hf coupling strength manifests itself in the spectral line shape. This change would be symmetric around the principal *g*-values of the detected radical as shown for the C_3, C_5 and ^{1}H-C*β*1 couplings.
The marginal deviation observed for in-cell samples due to the presence of Mn^{2+} is not symmetrical and is only observed at the low-field part of g_x. The spectral line shapes of in-cell and *in vitro* Y_{122}•s are otherwise identical. The Mn^{2+} background signals are attributed to the Mn^{2+} species inherently present in the cells (see SI 2.5 and 2.6). Distinct radical to Mn^{2+} concentration ratios in the two in-cell samples, as well as a small uncertainty in the microwave power lead to different Mn^{2+} intensities observed in the two in-cell traces (marked with * in Figure S4 B).

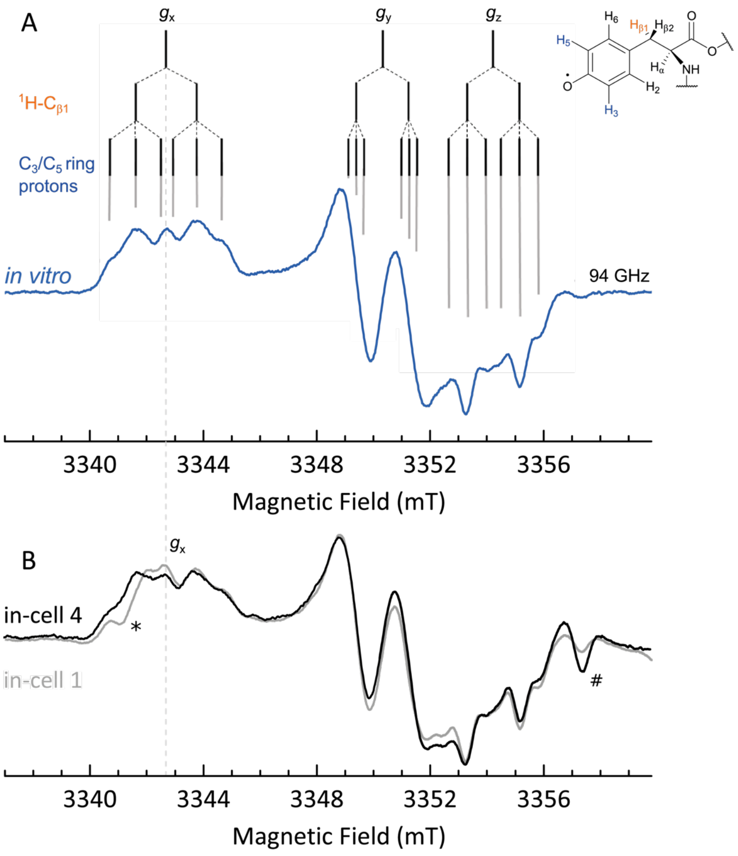

Figure S4. (A) First-derivative pulse EPR spectrum of an *in vitro* sample recorded at 94 GHz, shown together with its splitting scheme. Acquisition parameters are given in Table S1. (B) Direct comparison of two in-cell EPR spectra recorded at 94 GHz without background correction. Position of the g_x component is displayed with a grey vertical line. Spectral feature originating from Mn^{2+} in cells, which overlaps with the g_x region of Y_{122}•, is marked with an asterisk, * (see Figure S6). Difference in the EPR line shapes of the two in-cell samples observed in the g_x region is due to: i) a small difference in the microwave power for the two measurements, which strongly affected the Mn^{2+} intensity; ii) distinct radical to Mn^{2+} concentration ratios. An unidentified background signal observed both *in vitro* and in-cell and unrelated to Y_{122}• is marked with #.

2.5. Detection and evidence of Mn^{2+} species in the cellular environment

In order to display the contribution of the Mn^{2+} species to the spectra of in-cell sample, 9 Hahn-echo and 3 refocused echo (pump pulse off) experiments at 34 GHz were recorded with three different samples; 1) *in vitro* sample (240 μM Y_{122}• radical concentration), 2) in-cell sample 1 (22 μM Y_{122}• bulk radical concentration), and 3) *E. coli* cells lacking the β2 overexpression plasmid (0 μM Y_{122}• radical concentration). They were either optimized to detect Y_{122}• or Mn^{2+} at pump and/or detect frequencies of the DEER experiments. The optimization for the Mn^{2+} detection was performed by lowering the microwave power and shot repetition time. The six characteristic hyperfine lines of Mn^{2+} species are only visible in the in-cell and *E. coli* cells samples (see Figure S5). These experiments clearly demonstrate the huge contribution of Mn^{2+} species to the detected DEER signal at observer frequency. For the quantification of the Y_{122}• and Mn^{2+} contributions to the refocused echo with the pump pulse on, see SI sections 2.9 and 2.10.

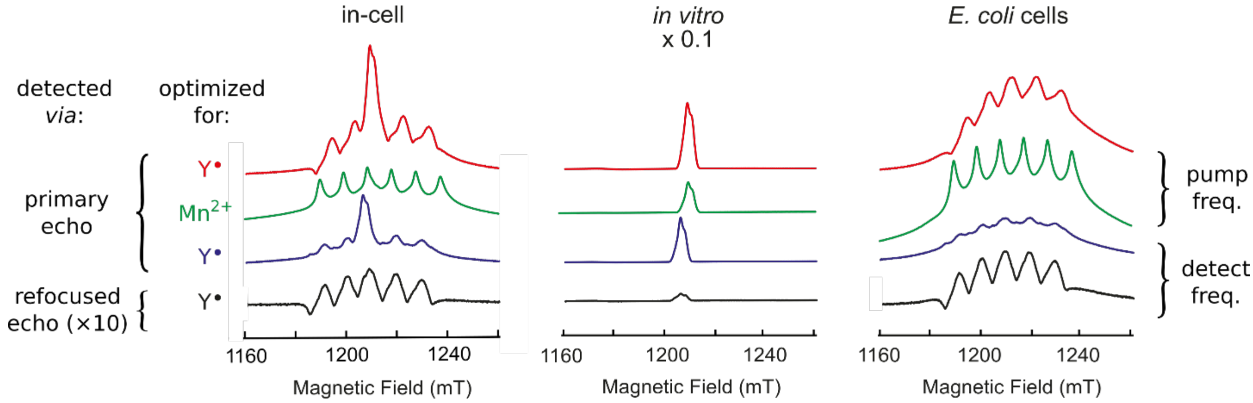

Figure S5. Pulse EPR spectra of Y_{122}• in in-cell (left) and *in vitro* (middle) samples compared to *E. coli* cells without overexpression plasmid (right). The detection is either optimized for Y• (red) or for Mn^{2+} (green). Furthermore, results of the optimization for Y• at the detect frequency (blue) are shown along with the refocused echo field-swept spectra (black). The EPR spectra were recorded at 10K and normalized to the same video gain and number of scans. Experimental conditions: t_π = 28 ns for the measurements at the pump frequency and t_π = 70 ns for the detect frequency; τ_1 = 300 ns (green with 200 ns), τ_2 = 2.1 µs (refocused echo); srt = 4 ms (green with 2 ms), shots per point = 100, microwave power = 20 mW (green with 3.2 mW).

2.6. Subtraction of the Mn^{2+} spectral features present in the EPR spectra of in-cell samples. Details of orientation-selective ^{1}H ENDOR spectra recorded at 34 GHz

In order to demonstrate that the Mn^{2+} species present in *E. coli* cells is the reason behind the marginal differences between the *in vivo* and *in vitro* EPR line shapes (especially at frequencies higher than 9.6 GHz), we recorded EPR spectra of *E. coli* cells without Y_{122}• at 34 and 94 GHz. The cells either lacked the overexpression plasmid or were harvested prior to the radical generation. Subsequently, we used these spectra for background correction of the in-cell data. The difference spectra are nearly identical to those recorded *in vitro* (orange *vs* green in Figure S6). These results clearly demonstrate that the only difference observed is due to the Mn^{2+} species inherently present in the cells.

In addition, the magnetic field positions corresponding to g_{xy}, g_y and g_{yz} molecular orientations used for orientation-selective ^{1}H Davies ENDOR measurements at 34 GHz are shown with vertical gray lines in Figure S6 (left).

For the W-band in-cell spectrum two types of background measurements had to be performed. The unidentified background peak labeled with #, as well the sharp hyperfine structure lines originating from the M_S = -1/2 ↔ +1/2 transition of Mn^{2+} were both detected for the *E. coli* cells harvested prior to the radical generation (solid magenta trace, Figure S6 right). However, the Mn^{2+} EPR intensity was strongly dependent on the microwave power. Since it was impossible to perfectly reproduce the same conditions for the background measurement, this background trace could only be used to isolate (dashed pink trace) and subsequently subtract the background peak labeled with #. To obtain a Mn^{2+} background, the in-cell spectrum was remeasured with a short shot repetition time, reduced by the factor of 30 (dark purple trace). This strongly suppressed the slowly relaxing Y_{122}• contribution, while reproducing the faster-relaxing Mn^{2+} background under the same conditions (microwave power and signal phase) as used for the in-cell Y_{122}• measurement. This trace was subsequently used to suppress the Mn^{2+} spectral features in the in-cell Y_{122}• spectrum.

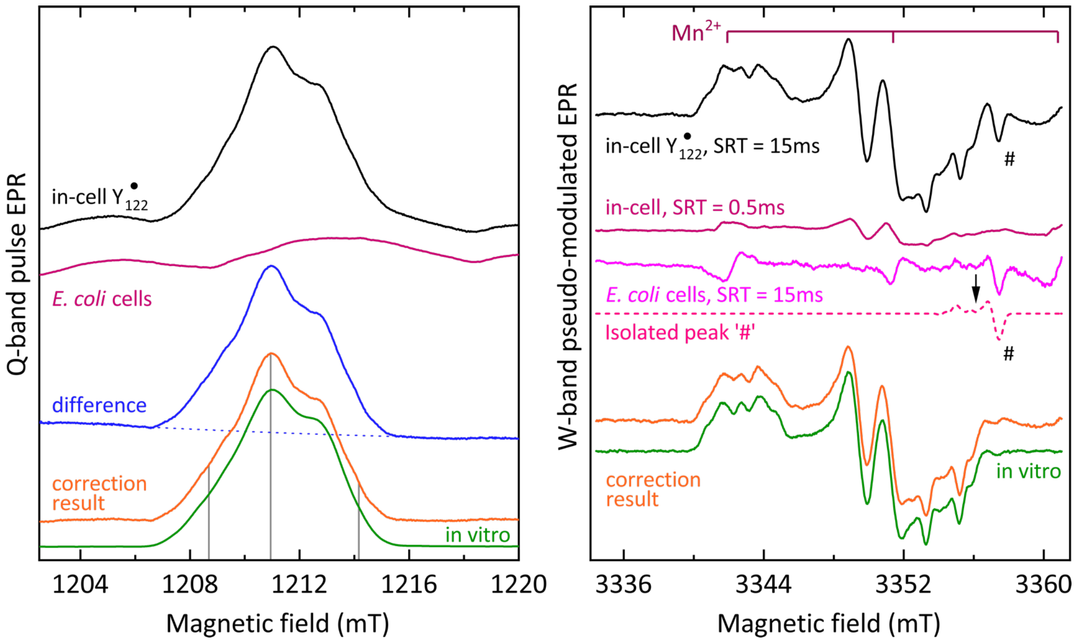

Figure S6. Background correction for the in-cell Y_{122}• EPR spectra detected *via* primary spin echo at Q-band (left) and W-band (right). **Q-band.** Black trace: EPR spectrum of Y_{122}• in whole *E. coli* cells recorded at 34 GHz, superposition of the Y_{122}• EPR line and broad, over-rotated Mn^{2+} features. Purple trace: spectrum of *E. coli* cells without the overexpression plasmid containing only endogenous radical below the detection limit, recorded under the same conditions. Blue trace: Y_{122}• line shape upon scaled subtraction of the Mn^{2+} background, shown together with a polynomial fit of the residual background (dashed blue line). Orange trace: background-corrected in-cell Y_{122}• EPR spectrum, with an *in vitro* Y_{122}• EPR spectrum (green trace) shown for comparison. Gray lines indicate the magnetic field positions of the orientation-selective ^{1}H Davies ENDOR measurements. **W-band.** Black trace: EPR spectrum of Y_{122}• in whole *E. coli* cells recorded at 94 GHz. Purple trace: spectrum of the same sample, with the Y_{122}• contribution suppressed by a short shot repetition time. Magenta trace: spectrum of *E. coli* cells harvested prior to the radical generation and containing only endogenous radical below the detection limit, recorded under approximately the same conditions as the in-cell Y_{122}• spectrum. Pink dashed line: isolated background signal labelled "#". Orange trace: background-corrected in-cell Y_{122}• EPR spectrum, with an *in vitro* Y_{122}• EPR spectrum (green trace) shown for comparison.

2.7. Details of the Y_{122}• DEER measurements and analysis

The DEER time trace, *V*(t), is a product of intermolecular contribution *B*(t) and intramolecular interaction *F*(t).[12] In order to separate *F*(t), which is the first step in analysis, *V*(t) is divided by *B*(t), which is commonly known as background function. Along with the background-corrected data, Fourier transform (FT) of *F*(t) is also shown below. In an ideal case, in which all the orientations are excited (no orientation selectivity), FT results in a spectrum called Pake pattern. DEER time traces of rigid spin pairs that are strongly correlated depend on the relative orientation of these spins, known as orientation selectivity. As our DEER experiments did not result in ideal Pake patterns, we recorded orientation-selective DEER experiments shown below in Figure S7. Additionally, two-pulse echo decay measurements were performed (Figure S8).

Parameters chosen for background correction procedure might result in unreal distance distribution peaks. DeerAnalysis2019 offers a validation tool to estimate the errors in the determination of the mean distances and their distributions. This tool calculates a distance distribution and carries out statistical analysis for a given set of parameters. Here, we calculated confidence intervals for in-cell and *in vitro* DEER traces by varying the starting values of the background fit by ± 50% in 10 steps and the background dimensionality in 10 steps by ± 0.5 with respect to the value chosen for the data analysis (shaded areas in Fig. S7). The detected mean distances with in-cell and *in vitro* samples were both validated.

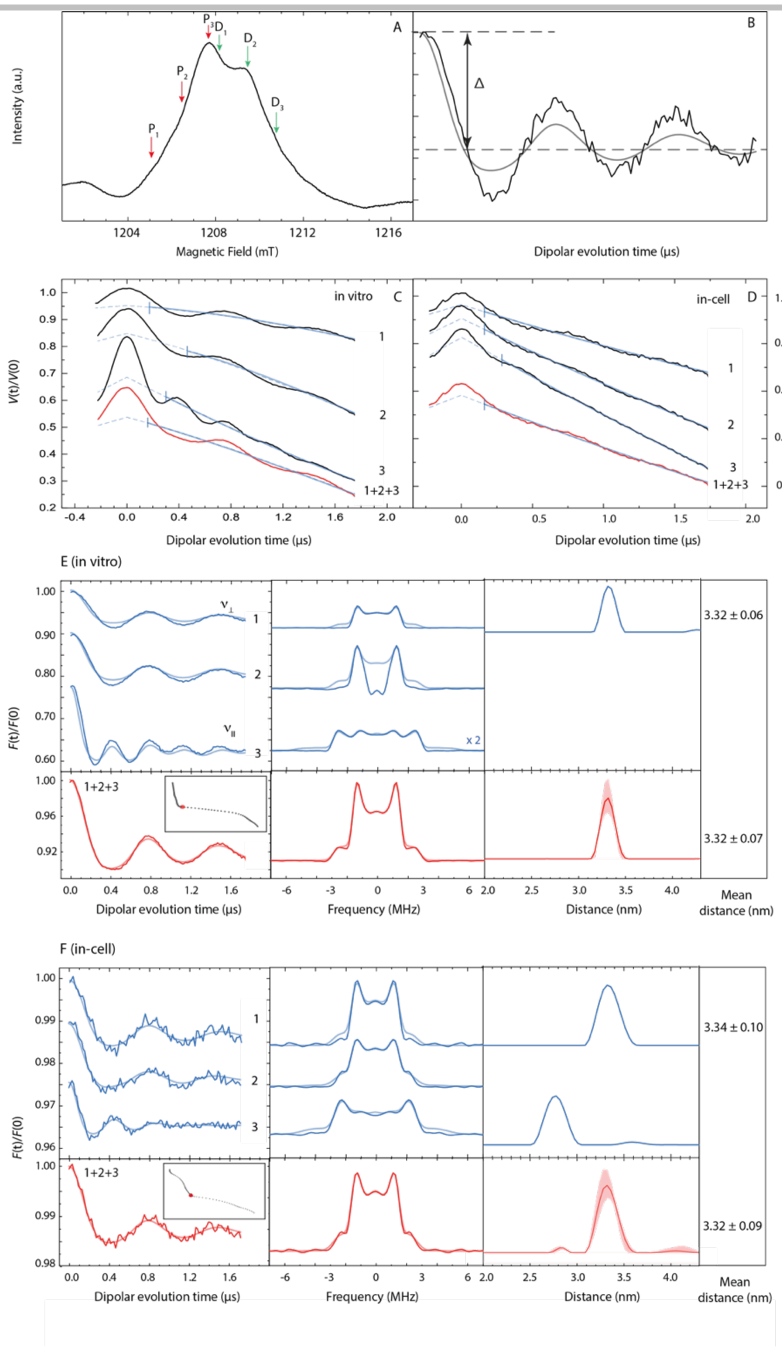

Figure S7. DEER measurements of Y_{122}• in *E. coli* RNR performed at 34 GHz and 10 K. **(A)** ESE-detected EPR spectrum of in-cell sample 1 recorded at 10 K. Three consecutive measurements for *in vitro* and in-cell samples were spaced by 14 G. Detect (D_1, D_2, and D_3) and pump (P_1, P_2, and P_3) positions for orientation-selective DEER measurements are displayed with green and red arrows, respectively. Frequency separations between detection and pump positions were 84 MHz. **(B)** Modulation depth parameter Δ is shown exemplarily using *in vitro* data. **(C)** Primary orientation-selective DEER traces (1, 2, and 3 shown with solid black lines) recorded with an *in vitro* sample shown along with the background functions (dashed blue lines). For orientation averaging, each primary time trace was first normalized to the same signal intensity at zero time. Afterwards, these traces were normalized to the signal intensity at the pump position. Summation led to the orientation-averaged time trace (red). **(D)** Primary DEER traces (1, 2, and 3 shown with solid black lines) recorded with the in-cell sample shown along with the background functions (dashed blue lines). Normalization was performed as described in (C). **(E)** Analysis of the *in vitro* DEER data at three different positions (upper row) along with the orientation-averaged time trace (lower row, red). **Left**: Background- and phase-corrected, normalized (time signal *V*(t) divided by the signal at echo maximum *V*(0)) DEER time traces are shown in dark colours. Fits obtained by DEERAnalysis2019[13] using Tikhonov regularization are overlaid and displayed in paler shade. L-curve and the corresponding regularization parameter (red data point) are shown in the inset. **Middle**: Fourier transforms of the DEER data (blue) and their fits (paler shade). **Right**: obtained Y_{122}•-Y_{122}• distance distributions, along with the mean distances and standard deviations. Validation of the distance distribution was obtained by varying the starting value of the background fit (vertical line in C) by ± 50% in 10 steps and the background dimensionality in 10 steps by ± 0.5 with respect to the initial value chosen for the data analysis. **(F)** Analysis performed as shown in (E) for the in-cell data. Experimental conditions: Gaussian pulses, $\pi_{detection}$ = 70 ns (D_1), 68 ns (D_2, D_3); π_{pump} = 28 ns (P_1, P_2, P_3); srt = 4 ms; spp = 100; acquisition time = 19 h (in-cell position 1), 19 h (in-cell position 2), 19 h (in-cell position 3), 3 h (*in vitro* position 1), 3h (*in vitro* position 2), 17 h (*in vitro* position 3).

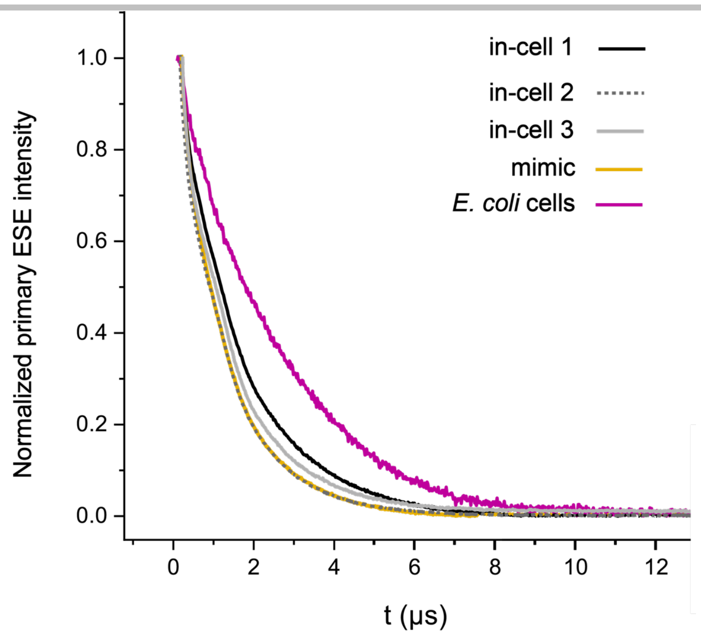

Figure S8. Two-pulse echo decay measurements of Y_{122}• in in-cell (black to grey) and *in vitro* mimic 1 with a relative Y_{122}• contribution of ~0.18 (yellow). Data for *E. coli* cells without overexpression plasmid (purple) are shown for comparison.

2.8. Origin of the second distance observed with in-cell sample

The analysis of the orientation-averaged in-cell DEER trace resulted in a second distance distribution peak that was validated by the validation tool of DeerAnalysis. The contribution of this distance, and thus the number of spin pairs (if real) resulting in this peak, is tiny as assessed by comparison of peak intensities of the distance distributions. We evaluated three possibilities as the origin of this peak, and subsequently suggest that it is an orientation-selection artifact. The three possibilities are;

1. *A distinct Y_{122}•-Y_{122}• distance:* It cannot be a Y_{122}•-Y_{122}• distance arising from conformationally distinct Y_{122}• pairs because we only detected one Y_{122}• conformation. As explained in SI 2.4, any conformational change of this radical regardless of its contribution to the spectrum would manifest itself in the spectral line shape of 94 GHz EPR spectrum as observed previously.[14]

2. *Mn^{2+}-Mn^{2+} distance:* As reported previously, Mn^{2+} can occupy the iron site in wt-β2 *in vitro*.[15] Considering the high Mn^{2+} content within the cells, we investigated a possible Mn^{2+}-Mn^{2+} distance. In this regard, we performed Mn^{2+} DEER experiments with *E. coli* cells that either contain the apo wt-β2 protein or lack the overexpression plasmid. As shown below in Figure S9, none of the experiments showed any dipolar modulations, and thus did not result in any distances. These data displayed that the detected distances throughout this work do not belong to a Mn^{2+}-Mn^{2+} pair, and are not affected by the presence of Mn^{2+} in the cells; however, presence of Mn^{2+} causes a reduction in modulation depth and in signal-to-noise ratio.

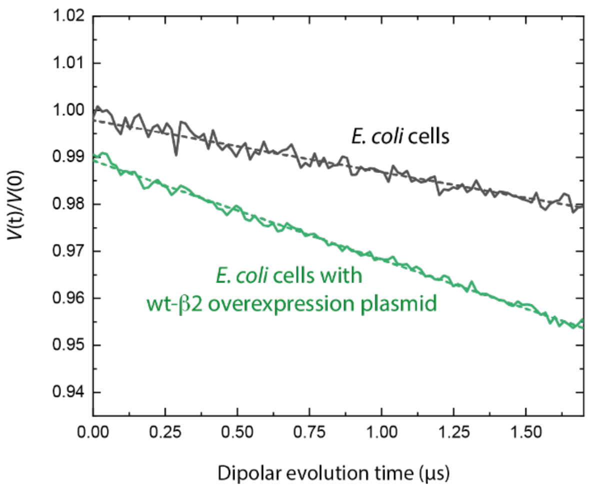

Figure S9. DEER measurements of *E. coli* cells that contain the wt-β2 overexpression plasmid (green) or lack it (black) at 34 GHz and 10 K. The fits that are shown with dashed lines are based on a homogenous, three-dimensional background function.

3. *Orientation selection artifact*: The observed second distance resembles the one extracted by DeerAnalysis when the distance vector $\boldsymbol{r}_{Y122\text{-}Y122}$ is parallel to the magnetic field, with the ν_{II} component dominating the dipolar spectrum (DEER time traces named '3' in Figure S7). For orientation-averaging, each primary time trace was first normalized to the same signal intensity at zero time. Afterwards, these traces were normalized to the signal intensity at the pump position. As the normalization of individual in-cell DEER traces was not ideal because of the poor signal-to-noise ratios (SNRs) and huge Mn^{2+} contribution at pump and detect positions, the distance resulting from the ν_{II} component might be over-pronounced in the orientation-averaged trace leading to the observed second peak. Indeed, during our experiments we realized that an improved SNR of the in-cell DEER trace recorded at D1 position led to a reduction of this second peak intensity in distance distribution analysis.

2.9. DEER data of three distinct in-cell samples

Three in-cell samples from distinct cell growths were prepared as explained in SI 1.1. Additionally, two *in vitro* samples were prepared to mimic in-cell samples having almost same Y_{122}• fractions (named mimic 1 and mimic 2). The details of spin concentrations of all samples are given in Table S2. Q-band field-swept EPR spectra of these samples recorded via refocused spin echo with the pump pulse applied at the primary echo position (normalized to the Mn^{2+} intensity), primary DEER time traces and their corresponding form factors are shown below in Figure S10. Note that even though background slopes of in-cell 2 and 3 are slightly steeper than that of in-cell 1, they do not exceed the slope of the 70 and 150 µM *in-vitro* samples shown in Figure 5B.

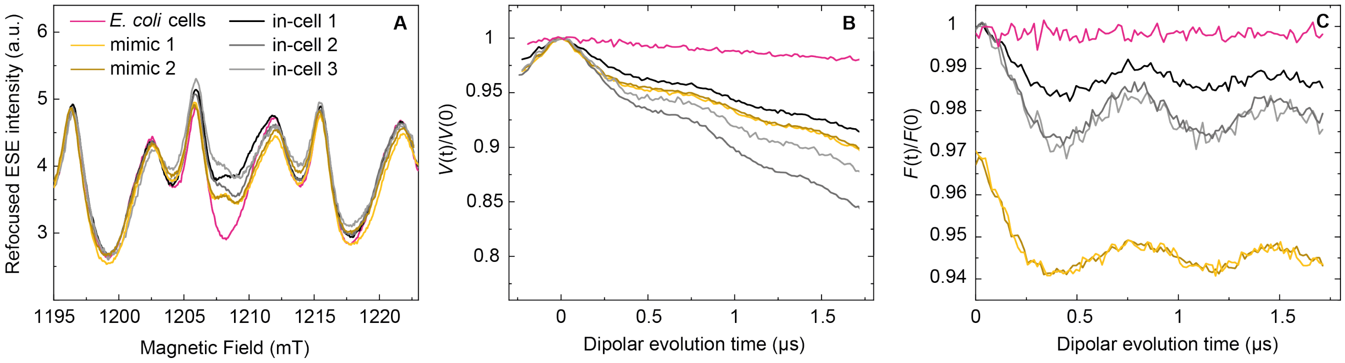

Figure S10. Modulation depth analysis of *in vitro* β2 mimic and in-cell samples in comparison to pure *E. coli* cells. (A) Q-band field-swept EPR spectra of *E. coli cells, in vitro* mimic duplicates and three different in-cell samples recorded via refocused spin echo as described in 2.10. (B) Primary DEER traces and (C) form factors of the corresponding samples (see S 2.11 for details of the background correction). Acquisition time: 15 h, 65 scans (*E. coli* cells); 14 h, 55 scans (mimic 1); 15 h, 62 scans (mimic 2); 19 h, 61 scans (in-cell 1); 22 h, 89 scans (in-cell 2); 25 h, 101 scans (in-cell 3).

2.10. Calibration curve for determining the expected modulation depths of in-cell samples

The DEER modulation depth is reduced by the presence of Mn^{2+} EPR signals overlapping with that of the radical. To create a calibration curve, *in vitro* samples containing different amounts of Y_{122}• and Mn^{2+} were prepared (at most 150 µM and 280 µM, respectively; see Table S2).

The relative contribution of the Y_{122}• signal for each sample was estimated via the refocused echo signal intensity recorded at the observe frequency. The primary echo intensity fractions of Mn^{2+} and Y_{122}• do not properly represent the corresponding values contributing to the DEER signal due to a shorter T_m time of Y_{122}• (the radical's relative contribution to the refocused echo intensity is reduced compared to that of Mn^{2+}). Furthermore, we found that switching on the π pump pulse optimized for an S = 1/2 species induces significant changes in the detected Mn^{2+} line shape (see Figure S11). The importance of the pump pulse influence on the Gd^{3+} refocused echo signal has been previously reported in the literature.[16] Thus, field-swept EPR spectra of several in-cell samples and various *in vitro* β2/Mn^{2+} mixtures were recorded by integrating the refocused echo with the pump pulse applied at zero dipolar time, with the same $\tau 1$ and $\tau 2$ delays as used in the DEER measurements. The detection window, as in DEER, was placed symmetrically around the maximum of the refocused echo of the S = 1/2 species.
In the next step, each field sweep was scaled to that of *E. coli* cells containing only Mn^{2+} and no Y_{122}• radicals (Fig. 5A and Fig. S10). Subtracting the *E. coli* cells EPR intensity, I(Mn^{2+}), at the observer position from that of the scaled in-cell and *in vitro* spectra, I(Mn^{2+}) + I(Y_{122}•), yielded the radical intensity, I(Y_{122}•). Then the relative contribution of the radical to the refocused echo intensity was obtained as I(Y_{122}•) / [I(Mn^{2+}) + I(Y_{122}•)]. The horizontal error gates were derived from the uncertainty in the background scaling procedure. The vertical error bars were derived from the uncertainty in the modulation depth determination.

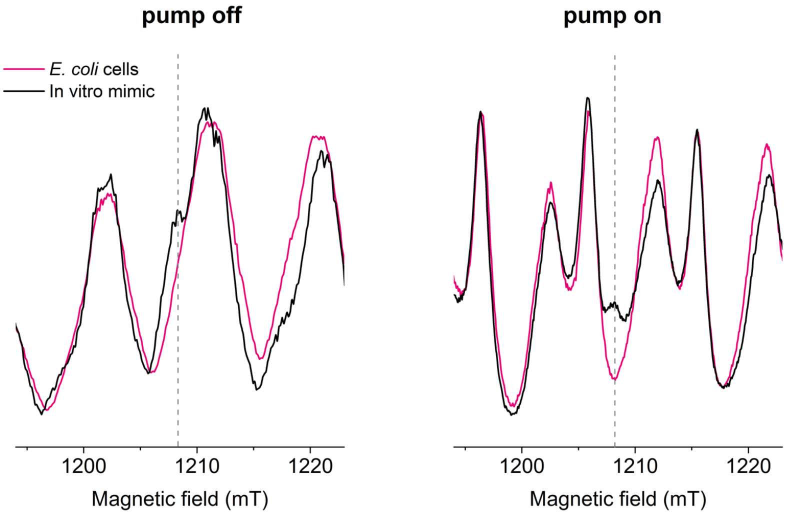

Figure S11. Refocused spin echo field sweep experiments for mimic 1 (black) and *E. coli* cells (purple) with the pump pulse off (left) or applied at the primary echo position (right). Spectra are normalized to the Mn^{2+} intensity. The observe position of the subsequent DEER experiment is marked with a dashed line. Experimental conditions: $\pi_{detection}$ = 70 ns, π_{pump} = 28 ns, srt = 4 ms; spp = 100, τ_1 = 300 ns, τ_2 = 2.1 µs, microwave power = 20 mW.

2.11. Background correction details

The DEER time traces were background corrected using second-order polynomial fitting (poly2) or homogeneous three-dimensional background correction (hom3). Both background functions resulted in almost identical distances and distributions (± 0.01 nm). However, the detected modulation depths were marginally different. We carefully compared the dipolar spectra resulting from distinct background functions for all the recorded traces. Poly2 consistently resulted in more reliable Pake patterns for all samples under investigation (see Figure S12A as an example). There are three possible reasons why poly2 was better for our samples:

1) Low modulation depth and SNR. This could be the reason for in-cell traces but not for the *in vitro* ones because the *in vitro* DEER traces display high SNRs.
2) A possible non-homogenous environment of the spins under investigation. Some examples of this kind are membrane proteins and/or pronounced aggregation of biomolecules.[17] A previous *in vitro* DEER study performed with *E. coli* RNR samples having different Y_{122}• concentrations showed that β2 dimers are homogenously distributed even at concentrations as high as 2.3 mM.[18] This concentration is ten times higher than that of our *in vitro* sample with the highest Y_{122}• concentration investigated here. Therefore, we concluded that aggregation of RNR β2 dimers is not the reason in our case.
3) The dipolar evolution time (spacing between the first Hahn echo and third detection pulse) of the DEER experiments is not long enough. A proper fitting can only be achieved when the dipolar evolution time in pulse sequence is significantly longer than the time required for dipolar modulations to fully decay. Unless the evolution time is set to extremely long values, the dipolar modulation observed with most of the RNR samples does not fully decay due to strong correlation between spins. The analysis of the long DEER traces shown in Figure S12B with both background functions resulted in highly similar Fourier transformed data; however, the hom3 has a deeper hole in the centre of the Pake pattern. This occurs when '*part of the biradical contribution is attributed to background*' (Jeschke, G., DeerAnalysis User Manual2013, https://epr.ethz.ch/software.html). Although we prolonged the dipolar evolution time as long as possible, dipolar modulations did not fully decay (lower trace in Figure S12B). We concluded that being unable to record traces whose dipolar modulations fully decay is the reason why poly2 resulted in more reliable Pake patterns in RNR samples. Additionally, poly2 has been the method of choice to analyze *in vitro* DEER data recorded with RNRs previously.[11a, 14, 19] In light of these results, we decided to employ the calibration curve obtained with poly2 in the main text. Note that the conclusion of presence of β2 dimers carrying only one Y_{122}• in the cells was also reached with the hom3 analysis, although poly2 and hom3 resulted in different modulation depth parameters, and thus slightly different calibration curves.

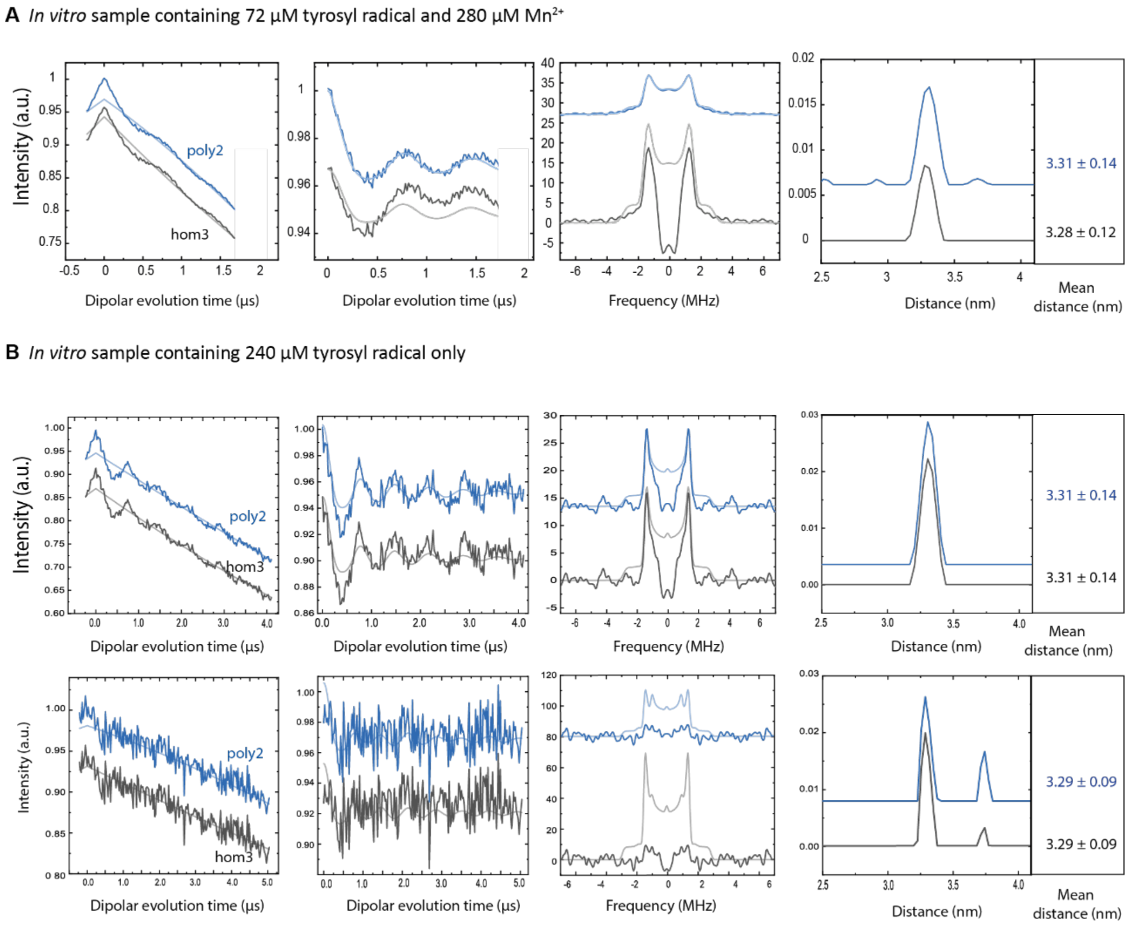

Figure S12. DEER traces of various *in vitro* wt-β2 samples with different dipolar evolution times. All traces were analyzed using either second-order polynomial fitting (poly2) or homogeneous three-dimensional background correction (hom3). For details of the analysis see SI 2.7. Acquisition time (A) 2h, 9 scans, (B) 22 h, 121scans (top) 42 h, 98 scans (bottom).

2.12. Concentration determination of the *in vitro* mimic sample

An *in vitro* sample containing 100 μM Mn^{2+} and an estimated Y_{122}• concentration of about 22 μM was prepared to mimic the in-cell sample (mimic 1). The radical concentration of this sample was estimated by comparing its 9.6 GHz cw-EPR intensity with those of *in vitro* (240 μM) and in-cell 1 (22 μM) as explained in S 2.3 (see Figure S13). These data demonstrated that mimic and in-cell samples contain almost identical amounts of Y_{122}•.

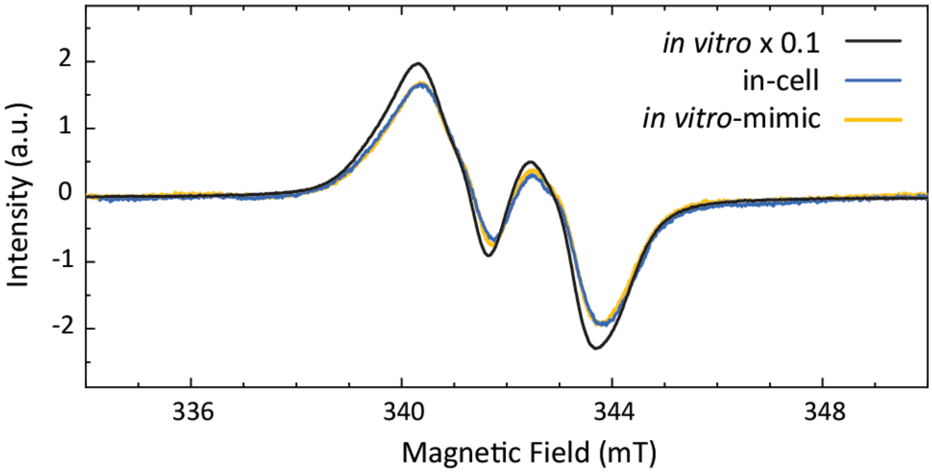

Figure S13. Determination of spin concentration via cw-EPR spectra of wt-β2 containing Y_{122}• recorded at 9.6 GHz and 100 K. Black: 240 μM purified RNR (*in vitro*). Blue: In-cell wt-β2 sample containing around 22 μM protein. Yellow: purified protein containing 22 μM Y_{122}• used to mimic the in-cell sample (mimic 1). Data were recorded at the Bruker EMXnano with 31.6 mW power, 1.5 G modulation amplitude, 100 kHz modulation frequency, 5.12 ms as time constant, and 19.9 ms conversion time; 20 scans (black), 200 scans (blue), 134 scans (yellow).

2.13. Detection of F_3Y_{122}• in whole *E. coli* cells

Comparison of 9.6 and 34 GHz supernatant, in-cell, and *in vitro* EPR spectra of F_3Y_{122}-β2 construct demonstrated that the EPR signal observed in the in-cell sample arises from the F_3Y_{122}• species residing in whole cells. Spectral simulations confirmed this result (Figure S14, Table S4).

Spin counting, which was performed by comparing the double integral of F_3Y_{122}• 9.6 GHz cw-EPR spectrum to that of the *in vitro* Y_{122}• reference, revealed bulk F_3Y_{122}• concentrations of 17 ± 5 μM and 130 ± 30 μM for in-cell and *in vitro,* respectively.

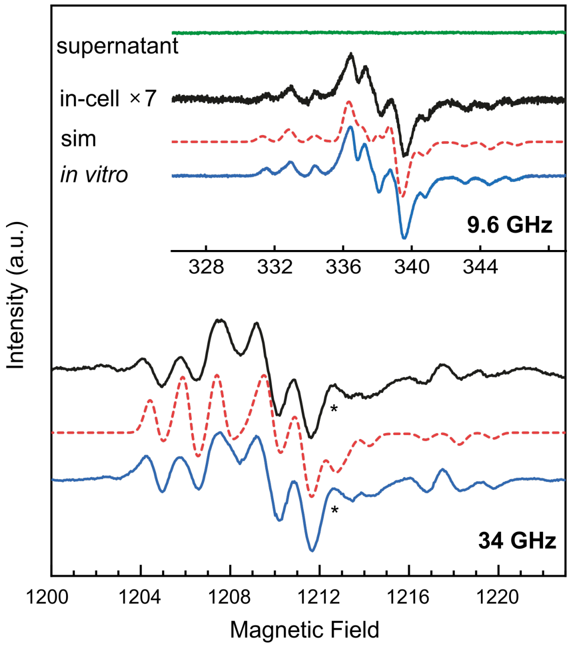

Figure S14. Cw- and derivative pulse EPR spectra of F_3Y_{122}• measured at 9.6 and 34 GHz. In-cell (black) and *in vitro* (blue) data are shown along with corresponding simulations (red dashed lines) and the supernatant spectrum (green). Simulation parameters reported in literature[4a, 20] are changed minimally during spectral simulations (see Table S4). Asterisks denote couplings seemingly absent in the experimental spectra due to a background signal. 9.6 GHz data were recorded at the Bruker EMXnano with 2.5 mW power (31.6 mW power for green), 1.5 G modulation amplitude, 100 kHz modulation frequency, 5.12 ms as time constant, and 19.9 ms conversion time at 100 K, 50 scans (black), 20 scans (blue), 100 scans (green). 34 GHz data were recorded with Bruker ELEXSYS-II E560 equipped with the 5106QT-2 resonator at 10 K. Experimental conditions: π = 32 ns, τ = 300 ns, srt = 4 ms, spp = 10 and 20 mW power, 1 scan.

Table S4. The simulation parameters reported in literature for *in vitro* F_3Y_{122}•[4a, 20] were changed in order to achieve the best fit. Best agreement with the experimental data was achieved with $g_{x,y,z}$ = 2.0085(5), 2.0045(2), 2.0022(3). Hyperfine parameters used for simulating the 9.6 GHz and 34 GHz data are given in regular and italic font, respectively. The small differences between the two sets are likely due to the presence of the second conformation. Euler angles α, ß, and γ are defined within the EasySpin z,y',z'' convention. They refer to rotations from the g-tensor frame into the hyperfine tensor frames. Positive angles are clockwise rotations viewed along the rotation axis. The hf coupling uncertainty was at most 20%.

		A_x / MHz	A_y / MHz	A_z / MHz	α	β	γ	*g* value
**This work**	$C_1\beta$-H	56<br>*56*	39<br>*39*	40<br>*40*				$g_{x,y,z}$ = 2.0085, 2.0045, 2.0022
	$C_2\beta$-H	0.5<br>*0.5*	0.5<br>*0.5*	3<br>*3*				
	F_2 ring	-3<br>*-3*	16<br>*16*	-45<br>*-45*	0	0	60	
	F_3 ring	-3<br>*-3*	-40<br>*-40*	150<br>*155*	0	0	-45	
	F_5 ring	-3<br>*-3*	-25<br>*-25*	180<br>*184*	0	0	135	
	H_6 ring	5.8<br>*5.8*	5.5<br>*5.5*	2<br>*2*	0	0	-120	
**Ref.[20]**	$C_1\beta$-H	56	39	40				$g_{x,y,z}$ = 2.0083, 2.0052, 2.0022
	$C_2\beta$-H	-	-	-				
	F_2 ring	-3	15	-45	0	0	60	
	F_3 ring	-3	-40	145	0	0	-42	
	F_5 ring	-3	-25	183	0	0	138	
	H_6 ring	7.8	7.5	2	0	0	-120	
**Ref.[4a]**	$C_1\beta$-H (IN conformation)	57	51	54				$g_{x,y,z}$ = 2.0082, 2.0051, 2.0021
	$C_1\beta$-H (OUT conformation)	43	38	38				
	$C_2\beta$-H (IN conformation)	0.5	0.5	3				
	$C_2\beta$-H (OUT conformation)	-0.5	-0.5	2				
	F_2 ring	8	-5	-37	0	0	120	
	F_3 ring	-15	-12	141	0	0	58	
	F_5 ring	-24	-16	187	0	0	-58	
	H_6 ring	11	8	7	0	0	-125	

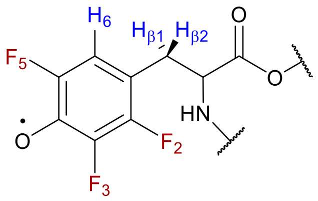

Scheme S2. Schematic representation of F_3Y_{122}• shown with the numbering of magnetically coupled nuclei included into the EPR spectral simulations in red and blue.

2.14. Details of the F_3Y_{122}• DEER measurements and analysis

As in section SI 2.7, we observed orientation selectivity with the *in vitro* sample, and thus performed orientation selection averaging by field stepping (Figure S15). Additionally, we calculated confidence intervals for in-cell and *in vitro* DEER traces by varying the starting values of the background fit by ± 50% in 10 steps and the background dimensionality in 10 steps by ± 0.5 with respect to the value chosen for the data analysis (see Fig. S15 for details). The detected mean distances for in-cell and *in vitro* samples were both validated.

The distance distribution obtained for *in vitro* F_3Y_{122}•-F_3Y_{122}• pairs is slightly larger than that observed for *in vitro* Y_{122}•-Y_{122}• pairs (0.11 vs. 0.07 nm). This result is in line with the F_3Y_{122}• having two different conformations (see Figure S16).

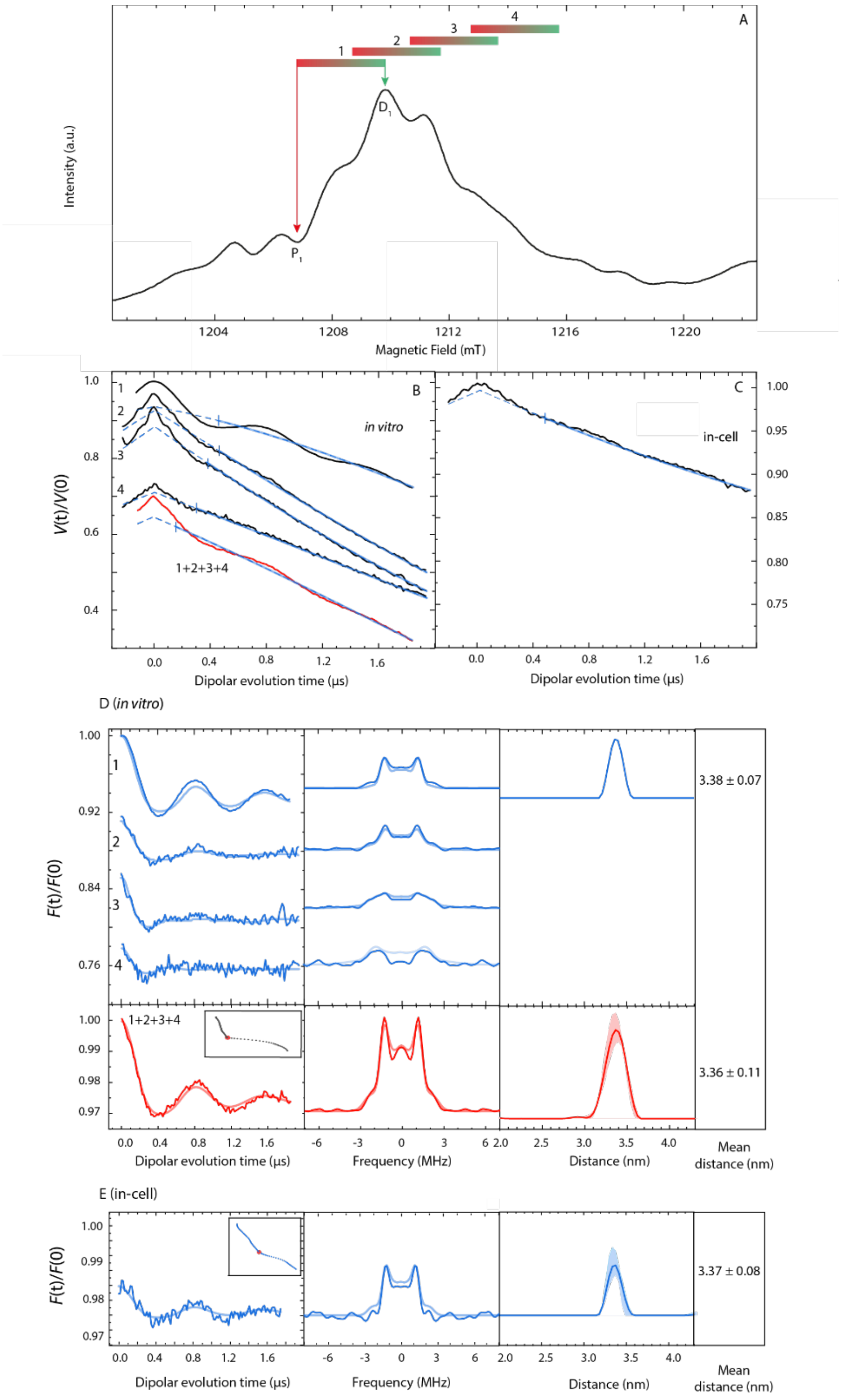

Figure S15. DEER measurements of F_3Y_{122}• in *E. coli* RNR performed at 34 GHz and 10 K. **(A)** ESE-detected EPR spectrum of an in-cell sample recorded at 10 K. Four consecutive measurements for an *in vitro* sample were spaced by 20 G. Detect (D_1, D_2, D_3, and D_4) and pump (P_1, P_2, P_3, and P_4) positions for *in vitro* orientation-selective DEER measurements are displayed with green and red marks, respectively. Frequency separations between detection and pump positions

were 84 MHz. DEER measurements for the in-cell sample were carried out at the first detect and pump position (D_1 /P_1). **(B)** Primary orientation-selective DEER traces (1, 2, 3, and 4 shown with solid black lines) recorded with the *in vitro* sample shown along with the background functions. For orientation averaging, each primary time trace was first normalized to the same signal intensity at zero time. Afterwards these traces were normalized to the signal intensity at the pump position. Summation led to the orientation-averaged time trace (red). **(C)** Primary DEER trace of the in-cell sample recorded at D_1/P_1 (solid black line) along with the background function (dashed blue line). **(D)** Analysis of the *in vitro* DEER data at four positions (upper row) along with the orientation-averaged time trace (lower row, red). **Left**: Background- and phase-corrected, normalized (time signal *V*(t) divided by the signal at echo maximum *V*(0)) DEER time traces are shown in dark colours. Fits obtained by DEERAnalysis2019[13] using Tikhonov regularization are overlaid and displayed in paler shade. L-curve and the corresponding regularization parameter (red data point) are shown in the inset. **Middle**: Fourier transforms of the DEER data along with their fits (paler shade). **Right**: Obtained F_3Y_{122}•-F_3Y_{122}• distance distributions, along with the mean distances and standard deviations. Validation of the distance distribution was obtained by varying the starting values of the background fit (vertical line in B) by ± 50% in 10 steps and the background dimensionality in 10 steps by ± 0.5 with respect to the initial value chosen for the data analysis. **(E)** Analysis performed as shown in (D) for the in-cell data (D_1/P_1 position). Experimental conditions: Gaussian pulses, $\pi_{detection}$ = 74 ns (D_1), 68 ns (D_2, D_3), 62 ns (D_4); π_{pump} = 32 ns (P_1, P_2, P_3, P_4); srt = 8 ms (in cell), 8 ms (*in vitro* position1), 4 ms (position 2-4); spp = 100; acquisition time = 44 h (in-cell), 10 h (*in vitro* position 1), 2.5 h (*in vitro* position 2), 4.5 h (*in vitro* position 3), 2 h (*in vitro* position 4).

2.15. F_3Y_{122}-F_3Y_{122} distance obtained from the crystal structure of F_3Y_{122}-β2

The crystal structure of F_3Y_{122}-β2 variant of *E. coli* RNR (PDB: 5CI3)[4a] was overlaid to the wt-β2 structure (PDB: 5CI4)[4a] to reproduce the homodimeric complex using PyMOL. In the crystal structure two conformations of F_3Y_{122} are present, the IN (yellow) and OUT (orange).[4a] The distance for IN conformers (3.36 nm) corresponds to the *in vitro* and in-cell distances detected via DEER (see Fig. S16). The F_3Y_{122}•-F_3Y_{122}• distance between two OUT conformers is 3.22 nm. Our results support that the IN conformation is predominant in whole cells as suggested in the literature.[4a]

Note that the IN-IN and OUT-OUT F_3Y_{122}•-F_3Y_{122}• distances are acquired between two oxygen atoms of F_3Y_{122}• in the crystal structure. The distances measured for a Y_{122}•-Y_{122}• pair based on the center of gravity of the spin density on Y•s[21] and based on the O atoms of Y•s in the crystal structure are 3.26 and 3.29 nm, respectively. Since this discrepancy is negligible (0.03 nm) compared to the difference between IN and OUT conformers (0.14 nm), and since it is estimated[4a] that the spin density distribution of F_3Y_{122}• remains roughly the same as that of Y_{122}•, we conclude that our distance calculation based on the oxygen atoms of F_3Y_{122}• in the crystal structure is valid.

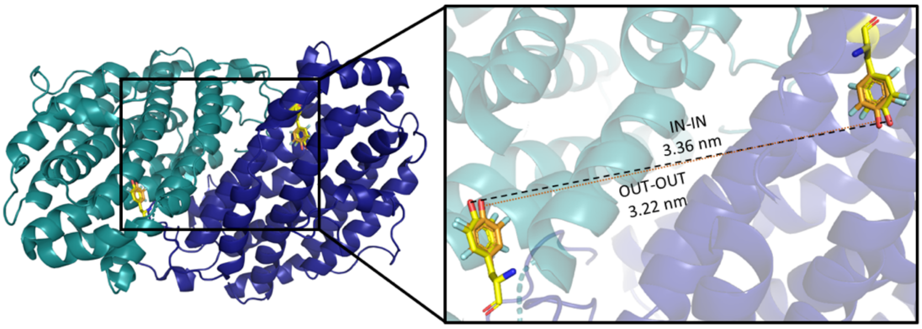

Figure S16. Cartoon representation of F_3Y_{122}-β2 variant of *E. coli* RNR.

References

[1] D. Hristova, C. H. Wu, W. Jiang, C. Krebs, J. Stubbe, *Biochemistry* **2008**, *47*, 3989-3999.
[2] a) J. M. Bollinger, Jr., W. H. Tong, N. Ravi, B. H. Huynh, D. E. Edmondson, J. A. Stubbe, *Methods Enzymol.* **1995**, *258*, 278-303; b) S. P. Salowe, J. Stubbe, *Journal of Bacteriology* **1986**, *165*, 363-366.
[3] N. Kalantari, S. Ghaffari, *Iran J. Environ. Healt.* **2008**, *5*, 173-178.
[4] a) P. H. Oyala, K. R. Ravichandran, M. A. Funk, P. A. Stucky, T. A. Stich, C. L. Drennan, R. D. Britt, J. Stubbe, *J. Am. Chem. Soc.* **2016**, *138*, 7951-7964; b) K. Yokoyama, U. Uhlin, J. Stubbe, *J. Am. Chem. Soc.* **2010**, *132*, 8385-8397.
[5] C. E. Tait, S. Stoll, *Phys. Chem. Chem. Phys.* **2016**, *18*, 18470-18485.
[6] M. Teucher, E. Bordignon, *J. Magn. Reson.* **2018**, *296*, 103-111.
[7] I. Ritsch, H. Hintz, G. Jeschke, A. Godt, M. Yulikov, *Phys. Chem. Chem. Phys.* **2019**, *21*, 9810-9830.
[8] I. Tkach, I. Bejenke, F. Hecker, A. Kehl, M. Kasanmascheff, I. Gromov, I. Prisecaru, P. Hofer, M. Hiller, M. Bennati, *J. Magn. Reson.* **2019**, *303*, 17-27.
[9] S. S. E. Gareth R. Eaton, David P. Barr, Ralph T. Weber, *Quantitative EPR, Vol. 1*, Springer-Verlag/Wien, **2010**.
[10] L. Wang, Y. J. Zhou, D. Ji, Z. K. Zhao, *J. Microbiol. Methods* **2013**, *93*, 73-76.
[11] a) M. Kasanmascheff, W. Lee, T. U. Nick, J. Stubbe, M. Bennati, *Chem. Sci.* **2016**, *7*, 2170-2178; b) H. M. McConnell, *J. Chem. Phys.* **1956**, *24*, 764-766.
[12] A. D. Milov, A. B. Ponomarev, Y. D. Tsvetkov, *Chem. Phys. Lett.* **1984**, *110*, 67-72.
[13] G. Jeschke, V. Chechik, P. Ionita, A. Godt, H. Zimmermann, J. Banham, C. R. Timmel, D. Hilger, H. Jung, *Appli. Magn. Reson.* **2006**, *30*, 473-498.
[14] T. U. Nick, K. R. Ravichandran, J. Stubbe, M. Kasanmascheff, M. Bennati, *Biochemistry* **2017**, *56*, 3647-3656.
[15] M. Atta, P. Nordlund, A. Aberg, H. Eklund, M. Fontecave, *J. Biol. Chem.* **1992**, *267*, 20682-20688.
[16] a) I. Kaminker, H. Yagi, T. Huber, A. Feintuch, G. Otting, D. Goldfarb, *Phys. Chem. Chem. Phys.* **2012**, *14*, 4355-4358; b) L. Garbuio, E. Bordignon, E. K. Brooks, W. L. Hubbell, G. Jeschke, M. Yulikov, *J. Phys. Chem. B* **2013**, *117*, 3145-3153; c) M. Yulikov, P. Lueders, M. Farooq Warsi, V. Chechik, G. Jeschke, *Phys. Chem. Chem. Phys.* **2012**, *14*, 10732-10746.
[17] G. Jeschke, *Annu. Rev. Phys. Chem.* **2012**, *63*, 419-446.
[18] M. Bennati, J. H. Robblee, V. Mugnaini, J. Stubbe, J. H. Freed, P. Borbat, *J. Am. Chem. Soc.* **2005**, *127*, 15014-15015.
[19] a) V. P. Denysenkov, T. F. Prisner, J. Stubbe, M. Bennati, *Proc. Natl. Acad. Sci.* **2006**, *103*, 13386-13390; b) J. Livada, R. J. Martinie, L. M. K. Dassama, C. Krebs, J. M. Bollinger, A. Silakov, *J. Phys. Chem. B* **2015**, *119*, 13777-13784.
[20] T. U. Nick, Hydrogen Bonds and Electrostatic Environment of Radical Intermediates in Ribonucleotide Reductase Ia, Georg-August-University Göttingen (Göttingen), **2015**.
[21] C. W. Hoganson, M. Sahlin, B.-M. Sjöberg, G. T. Babcock, *J. Am. Chem. Soc.* **1996**, *118*, 4672-4679.
